# Systematic Evaluation of Non-Uniform Sampling Parameters in the Targeted Analysis of Urine Metabolites by ^1^H,^1^H 2D NMR Spectroscopy

**DOI:** 10.1038/s41598-018-22541-0

**Published:** 2018-03-09

**Authors:** Trixi von Schlippenbach, Peter J. Oefner, Wolfram Gronwald

**Affiliations:** Institute of Functional Genomics, University of Regensburg, Am BioPark 9, 93053 Regensburg, Germany

## Abstract

Non-uniform sampling (NUS) allows the accelerated acquisition of multidimensional NMR spectra. The aim of this contribution was the systematic evaluation of the impact of various quantitative NUS parameters on the accuracy and precision of 2D NMR measurements of urinary metabolites. Urine aliquots spiked with varying concentrations (15.6–500.0 µM) of tryptophan, tyrosine, glutamine, glutamic acid, lactic acid, and threonine, which can only be resolved fully by 2D NMR, were used to assess the influence of the sampling scheme, reconstruction algorithm, amount of omitted data points, and seed value on the quantitative performance of NUS in ^1^H,^1^H-TOCSY and ^1^H,^1^H-COSY45 NMR spectroscopy. Sinusoidal Poisson-gap sampling and a compressed sensing approach employing the iterative re-weighted least squares method for spectral reconstruction allowed a 50% reduction in measurement time while maintaining sufficient quantitative accuracy and precision for both types of homonuclear 2D NMR spectroscopy. Together with other advances in instrument design, such as state-of-the-art cryogenic probes, use of 2D NMR spectroscopy in large biomedical cohort studies seems feasible.

## Introduction

Metabolomics aims at the comprehensive analysis of all metabolites in a biological system^1^. Nuclear magnetic resonance (NMR) spectroscopy is one of the most commonly used analytical techniques in metabolomics^2^, allowing the determination of a wide range of organic compounds in the millimolar to submicromolar range in a single measurement^3,4^. The analysis of biofluids is one of the main applications of metabolomics^5^. One of the most complex biological fluids in composition is urine, which typically contains hundreds of different solutes^6^ that can only be resolved in part by 1D ^1^H NMR. Multidimensional NMR spectroscopy, on the other hand, offers increased spectral resolution and, thus, reduced spectral overlap^5^. However, while 1D ^1^H NMR spectra are typically acquired in minutes, 2D NMR experiments may take several hours^7^. Such long acquisition times are impractical for large cohort studies and are not suited for unstable molecules. They may also cause spectral artefacts like *t*_1_ noise due to spectrometer instabilities^8^.

The experimental time needed for 2D NMR experiments to obtain sufficient digital resolution in the indirect dimension is dependent on the number of *t*_1_ increments collected^9^. Non-uniform sampling (NUS) acquires only a fraction of the indirect data points and reconstructs the spectra by non-Fourier methods, thus accelerating the acquisition of multidimensional NMR spectra^10,11^. The additional use of relaxation enhancing agents can even further expedite spectral data acquisition^12^.

In metabolomics, NUS has been primarily tested on standard mixtures of selected metabolites in often non-physiological concentration ranges^8–10^. Here, we used urine as a background matrix spiked with varying concentrations (15.6–500.0 µM) of tryptophan, tyrosine, glutamine, glutamic acid, lactic acid, and threonine to determine the impact of different sampling schemes, reconstruction algorithms and seed values on the fraction of indirect points that might be omitted without affecting the quantitative performance of both ^1^H,^1^H-TOCSY and ^1^H,^1^H-COSY45 experiments^13^. Finally, the best combination of acquisition parameters was applied to the determination of differences in urinary metabolite levels between apparently healthy subjects and patients suffering from chronic kidney disease (CKD).

## Results

**Figure 1:**
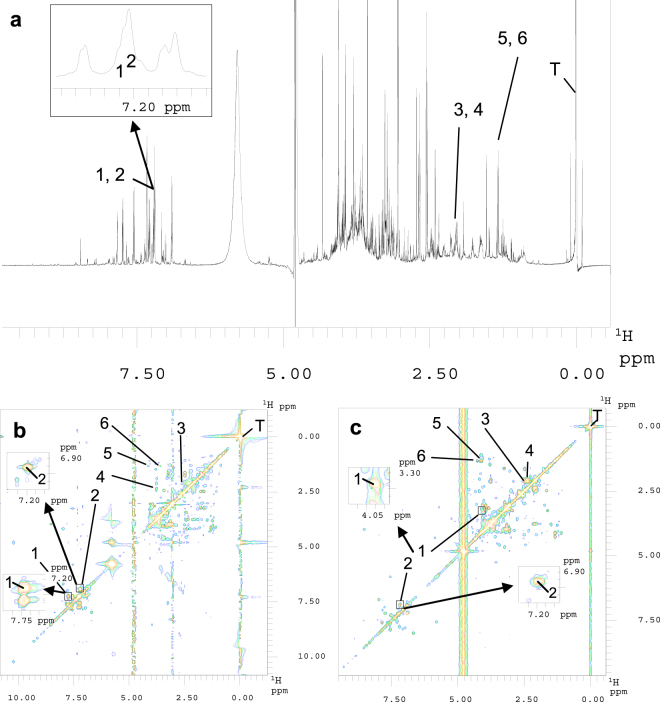
Exemplary NMR spectra of the human urine I specimen. Selected signals in the aromatic and aliphatic regions of the spectra were assigned to the six endogenous metabolites tryptophan (1), tyrosine (2), glutamine (3), glutamic acid (4), lactic acid (5), and threonine (6), which had been spiked-in at concentrations between 15.6 and 500.0 µM. While 1D ^1^H NMR spectroscopy failed to resolve the selected signals (**a**), both 2D ^1^H,^1^H-TOCSY (**b**) and ^1^H,^1^H-COSY45 (**c**) experiments yielded sufficient resolution as exemplarily shown for tryptophan and tyrosine via inserts. The contour levels of both 2D spectra are zoomed in close to the noise level. Key: T, 3-trimethylsilyl-2, 2, 3, 3-tetradeuteropropionate.

To test the impact of the choice of sampling scheme, reconstruction algorithm, and seed value used to initiate a pseudo-random number generator for setting the sequence of indirect data points to be collected^14^ we spiked a urine specimen (urine I) from an apparently healthy donor with varying concentrations of three pairs of urinary metabolites, namely tryptophan/tyrosine, glutamine/glutamic acid, and lactic acid/threonine, that had been chosen because of the inability of 1D ^1^H NMR spectroscopy to resolve them sufficiently for quantitation (Fig. 1a). Figures 1b and 1c show their successful spectral resolution by 2D ^1^H,^1^H-TOCSY and ^1^H,^1^H-COSY45 NMR spectroscopy, respectively.

### Reconstruction Algorithms

**Figure 2:**
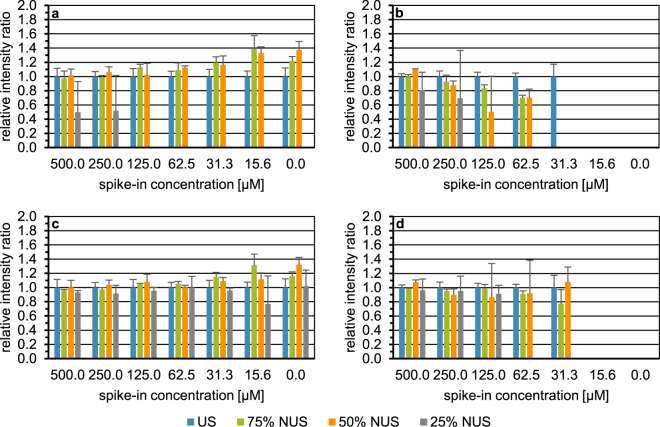
Impact of signal reconstruction with recursive multidimensional decomposition (**a**,**b**) and compressed sensing employing the iterative re-weighted least squares method (**c**,**d**), respectively, on the recovery of the relative intensity of a selected cross signal in the spike-in samples and the blank control employing urine I, exemplarily shown for glutamine (**a**,**c**) and glutamic acid (**b,d**). US ^1^H,^1^H-TOCSY spectra (*n* = 5) indicated by blue bars. NUS ^1^H,^1^H-TOCSY spectra (*n* = 3 each) measured with 75%, 50%, and 25% of the uniformly sampled data points depicted by green, orange, and grey bars, respectively. Each NUS spectrum was acquired with an exponentially weighted sampling scheme and three seed values per NUS level. The x-axis depicts the spike-in concentration in micromolar. The intensity ratio of the total cross peak integrals scaled to the internal standard 3-trimethylsilyl-2, 2, 3, 3-tetradeuteropropionate (TSP) of the metabolite signal (mean +SD) obtained with US or NUS to US is plotted on the y-axis.

We started with ^1^H,^1^H-TOCSY spectra to investigate the choice of either of the two different reconstruction algorithms implemented in TopSpin3.1 on the recovery of the six spiked-in urinary metabolites, as this type of spectra yields a larger number of signals than ^1^H,^1^H-COSY45 spectra, thus rendering reconstruction of NUS spectra more challenging. The two reconstruction algorithms initially tested were recursive multidimensional decomposition (R-MDD) and the compressed sensing approach employing the iterative re-weighted least squares method (CS-IRLS). Figures 2, S1, and S2 depict the recovery of metabolite signal intensities relative to uniform sampling (US) as a function of the level of sparse sampling (75%, 50%, and 25% of data points). The ratios serve as a measure of accuracy, whereas the coefficients of variation (CVs) reflect precision as exemplarily shown for glutamine (Fig. 2a–c) and glutamic acid (Fig. 2b–d). For the chosen levels of sparse sampling, separate bars are displayed for each spike-in level and the blank control. For the US ^1^H,^1^H-TOCSY spectra and for each level of sparse sampling, five and three spectra were acquired, respectively. The replicate spectra employing NUS were measured with an exponentially weighted sampling scheme and a different seed value for each replicate. To illustrate correlation trends between US and NUS, additional scatter plots with peak volumes normalised to TSP were generated (Fig. S3). For a NUS level of 25%, in particular, it is obvious that R-MDD failed more often than CS-IRLS to reconstruct signals at the lower spike-in concentrations (Figs 2, S1, and S2). Regarding the number of reconstructed signals per and across NUS levels (Table S1, first two columns), CS-IRLS always outperformed R-MDD. Note that a total of 40 signals were considered per NUS level, as one signal was selected per metabolite, each present at 6 different concentrations in the different spike-ins and the blank sample. For glutamic acid, only five signals were considered, as its signal intensities in the blank sample and at the lowest spike-in concentration were not present or too low for reliable integration even in the US ^1^H-^1^H TOCSY spectra. A Wilcoxon signed-rank test on the ratios and the coefficients of variation (CVs) across all spike-ins showed across all NUS levels significant differences between CS-IRLS and R-MDD (Table S2, first two columns) for both the ratios (*p* = 4.109*10^−6^) and CVs (*p* = 0.037). When comparing the mean ratios and CVs between CS-IRLS and R-MDD for each and across all NUS levels (first four columns of Table S3), CS-IRLS yielded more accurate and precise results than R-MDD. Taking all NUS levels into account, the mean ratio obtained by CS-IRLS was 0.96 with an average coefficient of variation (CV) of 12.30%, whereas the corresponding values for R-MDD were 0.89 and 17.16%, respectively. However, it is also obvious, that the performance of both reconstruction algorithms declined the less indirect data points were acquired, though in the case of the reconstruction of the 75% and 50% NUS spectra by CS-IRLS almost identical ratios of 0.98 and 0.99 were obtained. Overall, with regard to both the signals reconstructed and the accuracy and precision obtained, CS-IRLS proved superior to R-MDD. Therefore, CS-IRLS was used for further optimization.

### Sampling Schemes

Next, we investigated the influence of the three different sampling schemes and the choice of the seed value on the quantitative performance of ^1^H,^1^H-TOCSY experiments as a function of the fraction of indirect data points acquired. To assess the influence of the seed value regardless of the measurement error, simulated NUS spectra were generated by extracting data points of the indirect dimension from a single US spectrum in a fashion corresponding to an intended percentage of sparse sampling, seed value, and sampling scheme.

**Figure 3:**
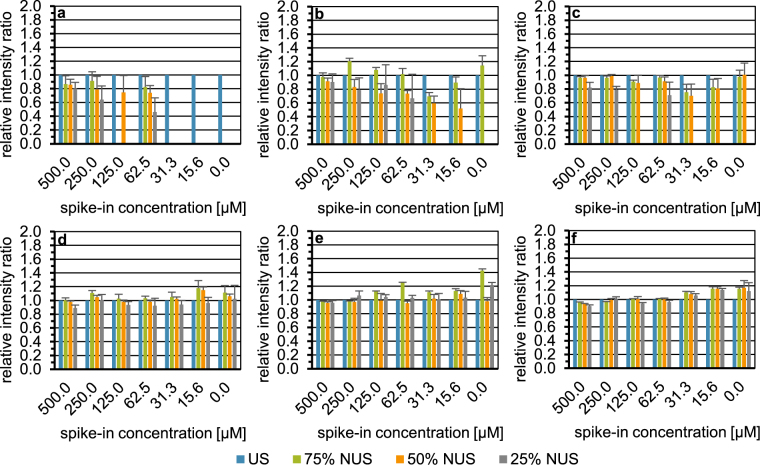
Influence of unweighted (**a**,**d**), exponentially weighted (**b**,**e**), and sinusoidal Poisson-gap (**c**,**f**) sampling on the recovery of the relative intensity of a selected cross signal in each spike-in and the blank employing urine I, exemplarily shown for lactic acid (**a**–**c**) and tyrosine (**d**–**f**). US ^1^H,^1^H-TOCSY spectrum (*n* = 1) indicated by blue bars. NUS ^1^H,^1^H-TOCSY spectra simulated from the US spectrum displayed with 75%, 50%, and 25% of the US data points depicted by green, orange, and grey bars, respectively. Each NUS spectrum was constructed with six seed values per NUS level and reconstructed with the compressed sensing approach employing the iterative re-weighted least squares method. On the x-axis, the spike-in concentration is given in micromolar. The intensity ratio of the total cross peak integral scaled to the internal standard TSP of the metabolite signal (mean +SD) obtained with US or NUS to US is plotted on the y-axis.

Figure 3 shows exemplarily for lactic acid (a–c) and tyrosine (d–f) the influence of an unweighted (a,d), exponentially weighted (b,e), and sinusoidal Poisson-gap (c,f) sampling scheme on the recovery of signal intensities relative to US. For each NUS level (75%, 50%, and 25%), six different seed values were employed for data extraction from the US spectrum. All NUS spectra were reconstructed by CS-IRLS. Data from Figs 3, S4, S5, and S6 are also depicted as scatter plots (Fig. S7). Of the three sampling schemes, sinusoidal Poisson-gap sampling performed best. While the tyrosine signal could be reconstructed with good accuracy by each of the sampling schemes, unweighted sampling failed to reconstruct the lactic acid signal at spike-in concentrations below 250 µM. Further, only sinusoidal Poisson-gap sampling allowed quantification of lactic acid in the blank employing 50% NUS.

Considering the number of recoveries observed over all NUS levels with either sampling scheme (Table S1), it is evident that the sine-weighted Poisson-gap sampling scheme recovered the most signals. The same is true for 50% NUS, while for 75% and 25% NUS Poisson-gap and exponentially weighted sampling performed equally well. Looking at the ratios (accuracies) over all spike-in metabolites and concentrations, the Friedman test (Table S2) showed significant differences across NUS levels (*p* = 2.536*10^−8^). Subsequent application of the Nemenyi post hoc test (Table S4) showed that both exponential weighted and sinusoidal Poisson-gap sampling differed significantly from unweighted sampling, while the former two did not differ significantly. Regarding ratios across all NUS levels (Table S3), the three sampling schemes differ little in accuracy, with unweighted, exponentially weighted, sinusoidal Poisson-gap sampling yielding average ratios of 0.97, 1.02, and 1.03, respectively. Regarding precision, the Friedman test (Table S2) shows a significant difference between the three sampling schemes over all NUS levels (*p* < 2.2*10^−16^). The Nemenyi post hoc test (Table S4) revealed no significant difference between unweighted and exponentially weighted sampling. In terms of precision significant differences, however, were observed between sinusoidal Poisson-gap and the other two sampling schemes (last two columns of Table S4). Results given in Table S3 demonstrate, that precision (CV) depends on the chosen sampling scheme, with the sinusoidal Poisson-gap sampling scheme depending the least on the seed value chosen, showing a CV of 5.39% over all NUS levels compared to unweighted and exponentially weighted sampling with a CV of 12.06% and 9.59%, respectively. In light of the greater number of signals recovered and the lowest seed value dependency, sine-weighted Poisson-gap sampling was used for all further evaluations and applications of NUS.

### Spectra types

**Figure 4:**
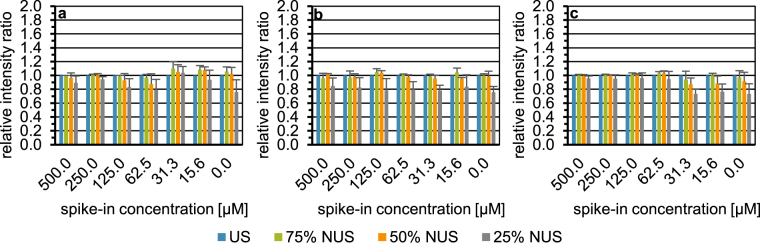
Influence of the spectral type on the recovery of the relative intensity of a selected cross signal in each spike-in sample and the blank employing urine I, exemplarily shown for tyrosine (**a**), glutamine (**b**), and lactic acid (**c**). US ^1^H,^1^H-COSY45 spectrum (*n* = 1) indicated by blue bars. NUS ^1^H,^1^H-COSY45 spectra simulated from the US spectrum displayed with 75%, 50%, and 25% of the US data points depicted by green, orange, and grey bars, respectively. Each NUS spectrum was constructed with a sinusoidal Poisson-gap sampling scheme taking six seed values per NUS level and reconstructed with the compressed sensing approach employing the iterative re-weighted least squares method. On the x-axis, the spike-in concentration given in micromolar is shown. The intensity ratio of the total cross peak integral scaled to the internal standard TSP of the metabolite signal (mean +SD) obtained with US or NUS relative to US is plotted on the y-axis.

To investigate a potential differential impact of NUS on the type of homonuclear 2D experiment used, ^1^H,^1^H-TOCSY spectra were compared to ^1^H,^1^H-COSY45 spectra. Figure 4 shows exemplarily the recovery of the tyrosine (a), glutamine (b), and lactic acid (c) signals in ^1^H,^1^H-COSY45 spectra, which shared the same cross signal positions in ^1^H,^1^H-TOCSY spectra. Results for the other three spike-in metabolites are depicted in Figure S8. Note that sine-weighted Poisson-gap sampling in combination with CS-IRLS was used here. Data from Figs 4 and S8 are also displayed as scatter plots (Fig. S9).

When comparing columns five and six in Table S1 and Figs 4, S6 and S8, the number of signals reconstructed successfully over all NUS levels is higher for ^1^H,^1^H-COSY45 than ^1^H,^1^H TOCSY. For both 75% and 50% NUS both spectra types showed a recovery of all 40 considered signals, whereas for 25% NUS 33 and 38 signals were recovered for ^1^H,^1^H TOCSY and ^1^H,^1^H-COSY45, respectively. When considering accuracy and precision, Table S2 shows that over all NUS levels significant differences were obtained between the two spectra types using the Wilcoxon signed-rank test. Next, for each NUS level, mean ratios and CVs were compared separately (the last two columns of Table S3 for ^1^H,^1^H-TOCSY and Table S5 for ^1^H,^1^H-COSY45). Results indicate that at NUS levels of 75% and 50%, ^1^H,^1^H-TOCSY and ^1^H,^1^H-COSY45 yield a similar accuracy with ratios of 1.06 and 1.02 at 75% NUS and of 1.05 and 0.96 at 50% NUS, respectively. At 25% NUS, in contrast, a considerable drop in accuracy was observed for ^1^H,^1^H-COSY45 spectra with the ratio to US decreasing to 0.80. In terms of precision, ^1^H,^1^H-TOCSY was more precise than ^1^H,^1^H-COSY45 with CVs of 5.39% and 8.89%, respectively, across the three NUS levels. However, the differences in accuracy and precision between ^1^H,^1^H-TOCSY and ^1^H,^1^H-COSY45 are mostly observed at the 25% NUS level. Applying the Friedman test, significant differences in accuracy and precision were observed across the different NUS levels in ^1^H,^1^H-COSY45 spectra (*p* < 2.2*10^−16^ and *p* = 4.0*10^−6^, respectively) (Table S6). In terms of accuracy the Nemenyi post hoc test showed significant differences for each NUS level comparison. The same is true in terms of precision except for the comparison of 75% against 50% NUS (Table S6).

In conclusion, ^1^H,^1^H-TOCSY or ^1^H,^1^H-COSY45 appear to perform equally well with regard to the number of signals reconstructed, accuracy, and precision as long as the fraction of data points acquired does not drop below 50%. However, ^1^H,^1^H-COSY45 is the more simple pulse sequence and, therefore, may be better suited for large metabolomic studies. The number of expected signals per metabolite is generally lower in ^1^H,^1^H-COSY45 than in ^1^H,^1^H-TOCSY spectra, which reduces signal overlap in highly complex biofluids such as human urine. Therefore, ^1^H,^1^H-COSY45 spectra have in this context a slight advantage over ^1^H,^1^H-TOCSY spectra.

Having shown that a sine-weighted Poisson-gap sampling scheme in combination with 50% NUS performed best on our experimental setup, we chose to evaluate two other commonly applied reconstruction algorithms, namely the Iterative Soft Thresholding Compressed Sensing method (CS-IST) implemented in the MestReNova software suite and the maximum entropy (MaxEnt) approach implemented in the Rowland NMR toolkit. CS-IST was evaluated on ^1^H,^1^HCOSY45 spectra acquired in magnitude mode as these spectra provided a slight advantage over phase sensitive ^1^H,^1^H-TOCSY spectra. As reconstruction by the applied MaxEnt approach is restricted to phase-sensitive data MaxEnt reconstruction was investigated on ^1^H,^1^H-TOCSY spectra. When comparing the number of signals reconstructed with the so far best performing method CS-IRLS, with CS-IST, and MaxEnt in Figs 4 and S8, S10, and S11, respectively, as well as in Table S7, CS-IRLS outperforms the other two reconstruction algorithms. This is particularly true for MaxEnt which is able to recover merely 31 out of all possible 42 signals compared to CS-IRLS yielding nearly all observable recoveries (Table S7). Recoveries obtained from data having applied MaxEnt show heavy underestimation for tryptophan, glutamic acid, lactic acid, and threonine and on the other hand strong overestimation of tyrosine signal volumes (Fig. S11). Results showed that CS-IRLS allows a more accurate and precise quantitation with a mean ratio of 0.96 and a CV of 8.01% in comparison to CS-IST with a mean ratio of 0.93 and a CV of 8.47% (Table S8). The difference is even most striking when comparing to MaxEnt showing a mean ratio of 0.61 and a CV of 13.70% (Table S8). Table S9 shows that significant differences in ratios but non-significant differences in CV were obtained between the three reconstruction algorithms having applied the Friedman test. Results from the Nemenyi post-hoc test show that significant differences in ratio are derived from results obtained from data reconstructed with MaxEnt (Table S10).

### Metabolites

Next, applying 50% NUS on ^1^H,^1^H-COSY45 spectra with a sinusoidal Poisson-gap sampling scheme and spectral reconstruction by CS-IRLS, we tested for metabolite-dependent differences in accuracy and precision. Table S11 lists the median accuracies and mean CVs of a given spike-in metabolite over all concentrations and per spike-in concentration, respectively. All metabolites yield acceptable accuracy and precision for the upper four spike-in concentrations, with tyrosine, glutamine, and lactic acid even yielding adequate accuracy and precision for the lower spike-in levels. All metabolites show on average, according to FDA guidelines, acceptable accuracies and precisions. Applying a Kruskal-Wallis test on accuracies (*p* = 0.0533) and a one-way ANOVA on the CVs (*p* = 0.1359), for the latter showing homogeneity of variances with the Bartlett test (*p* = 0.1502), no significant differences can be seen between the metabolites.

### Fold Changes

Finally, employing urine I the determination of fold changes was investigated. For each metabolite the difference between expected and observed fold changes was analysed. Note that before calculation of fold changes the corresponding signal intensities of the blank were subtracted from the spike-in data. Figure S12a summarizes the results for the *n* = 5 fold changes of two. For Figure S12b, all fold changes between 2 and 32 were considered to compute 15 fold changes of two. For reasons of comparison, also fold changes obtained from the corresponding US spectrum were included. For expected fold changes of two (Fig. S12a), the mean observed fold change over all metabolites was 2.08 for US, while it was 2.15 for 50% NUS showing an average error for both US and 50% NUS of less than 10%. Over all metabolites and six spectral replicates, 50% NUS has an acceptable precision according to FDA guidelines with a mean precision of 9.26%. Further, with the exception of lactic acid that yielded a CV of 22.52% in the 50% NUS data, the expected fold changes of two can be determined with an error in accuracy and precision of less than 15%. A paired Student’s *t*-test applied over all metabolites showed no significant differences in the observed fold changes between US and 50% NUS (*p* = 0.0666). Consideration of all fold changes of 2 to 32 (Fig. S12b) results in stronger deviations in accuracy and precision for all metabolites. For expected fold changes of 2 to 32, Figure S12c depicts the linear dependency between expected and observed fold changes over all spike-in metabolites in urine I as a further measure to assess the reproducibility of determining fold changes. The regression lines of both the US and 50% NUS dataset nearly overlap, each with a slope of about one, an offset near zero, and coefficients of determination of 0.999. Stronger variations of the observed fold changes from the expected fold changes determined with 50% NUS compared to US can be seen in the wider standard deviation ranges for 50% NUS.

### Parameter Validation

So far parameters were optimized based on one spike-in dataset. For validation purposes, a second spike-in dataset (urine II) was generated in the same manner as the first one except that this time a more concentrated urine matrix was used. As described above, for each sample a US ^1^H,^1^H-COSY45 spectra was acquired and reconstructed *in silico* employing the optimized set of parameters for 50% NUS. Table S12 lists the median accuracies and mean CVs of a given spike-in metabolite over all concentrations and per spike-in concentration, respectively. With the exception of tryptophan all metabolites yield acceptable accuracies and precisions for all spike-in concentrations. For the lower spike-in concentrations of tryptophan deviations in both accuracy and precision that exceeded FDA recommendations were observed. This is explained by the comparatively weak tryptophan signals. Overall, results from the two spike-in datasets agree quite well. Thus, it is concluded that the set of optimized parameters is applicable to different urinary matrices.

### Application of Accelerated Quantification with NUS to Urine Specimens

Next, 1D ^1^H NOESY and 2D ^1^H,^1^H-COSY45 spectra with and without 50% NUS were acquired for 28 urine specimens each selected at random from the German Chronic Kidney Disease (GCKD) Study and the German National Cohort (GNC). A short description of both studies is given in the Methods section. To compare the performance of the three types of NMR experiments, Bland-Altman plots were generated for a small selected set of metabolites of medical relevance in chronic kidney disease, namely creatinine, hippurate, lactate, D-glucose, citrate, glutamine and pseudouridine (Figs S13, S14, and S15). In Figs S14 and S15, the spike-in metabolites were considered in addition except for glutamic acid, which could not be detected in any of the GCKD and GNC urine specimens. The Bland-Altman plots depicted in Fig. S13 reveal no bias in the concentrations of creatinine (a), hippuric acid (b), and glucose (d) determined by 1D ^1^H NOESY and 50% NUS 2D ^1^H,^1^H-COSY45. In case of lactic acid (**c**), 1D ^1^H NOESY yielded increasingly higher concentrations compared to 50% NUS 2D ^1^H,^1^H-COSY45 with increasing urinary levels of lactic acid, while for citric acid (e) and glutamine (f) a considerable widening trend of the agreement range was observed with increasing concentrations of both metabolites. For the comparison of 2D ^1^H,^1^H-COSY45 data acquired by US and 50% NUS, respectively, no systematic bias is obvious and with the exception of tryptophan only minimal variations are observed for any of the nine metabolites investigated (Figs S14 and S15a). The agreement in urinary cohort sample metabolite levels determined by 50% NUS and US ^1^H,^1^H-COSY45 NMR spectroscopy, respectively, is furthermore demonstrated in the boxplots depicted in Figures S15b and S15c, S16, and S17, which show the levels of glutamine, hippuric acid, lactic acid, D-glucose, citric acid, and pseudouridine, respectively, normalised against the respective creatinine level in each specimen, for the two cohorts. Using a Mann-Whitney *U*-test, significant differences in the urinary levels of pseudouridine, citric acid, and glutamine between the two cohorts were observed (Table S13), with the average amount of pseudouridine being higher in the CKD specimens, while the opposite applied to glutamine and citric acid. Further, while the lower limits of quantification (LLOQs) for lactic acid, D-glucose, and glutamine did not differ between 50% NUS and US, the latter yielded two-fold lower LLOQs for hippuric acid, citric acid, and pseudouridine, and in case of creatinine the LLOQs differed even by a factor of 4 (Table S14). In comparison, the corresponding LLOQs for 1D ^1^H spectra are around 3 µM with the exception of glutamine, which yielded an LLOQ of 39 µM.

## Discussion

In this contribution, we have tested a number of combinations of sampling scheme, reconstruction algorithm, NUS level, and spectra type. It is clear that the combinations tested are by no means exhaustive and, therefore, it is possible that other combinations will give similar or even better results. With regard to the initiatory evaluation of NUS on the spike-in dataset, we find that, especially for the reconstruction of weak signals, CS-IRLS is clearly superior to both R-MDD and MaxEnt, whereas it performed slightly better than CS-IST. However, the strategies applied to date to the reconstruction of NUS spectra are versatile^15^ and differ in their assumptions about the properties of the time domain signal or reconstructed spectrum^16,17^. Assumptions applicable for NUS processing include the introduction of a certain model of a spectrum as exploited in MDD^17^, a spectrum containing the least amount of information consistent with the measured data as assumed in maximum entropy (MaxEnt)^18,19^ and in forward maximum entropy (FM) reconstruction^20^, knowledge on empty regions in a spectrum^21^ and maximum sparsity underlying compressed sensing (CS) approaches. Typical methods applied in the context of CS include iterative re-weighted least squares (IRLS)^22^, iterative soft thresholding (IST)^22^, orthogonal matching pursuit (OMP)^22^, its predecessor CLEAN^22^, and SCRUB^23^. Reconstruction algorithms appropriate for NUS are commonly non-parametric signal processing methods. However, parametric approaches such as maximum likelihood and Bayesian methods, where the time-domain signal is described as a sum of exponentially decaying sinusoids, have been also used^24^. Another suitable parametric method is the sparse multidimensional iterative lineshape enhanced (SMILE) algorithm, which integrates a priori information about NMR signals for robust signal reconstruction^25^. In order to extend our evaluation of the impact of the chosen reconstruction method on quantitative NUS data, we did not limit the applied reconstruction algorithms to the two implemented in TopSpin 3.1, R-MDD and CS-IRLS, but additionally implemented MaxEnt and CS-IST. We chose the latter two algorithms because MaxEnt is known to be a well established, very robust and versatile non-Fourier method which efficiently reduces artefacts^15,18,19,26,27^ and CS-IST is a popular CS-based approach next to CS-IRLS^22^. In order to keep the other NUS parameters comparable, simulated data were chosen for analysis applying a sinusoidal Poisson-gap sampling and a 50% sampling density. MaxEnt was applied to ^1^H,^1^H-TOCSY spectra because of the need for phase-sensitive data^20^ and CS-IST on magnitude mode ^1^H,^1^H-COSY45 spectra as they provided a slight advantage over ^1^H,^1^H-TOCSY spectra. Our data show that neither of these two reconstruction algorithms outperforms CS-IRLS. Findings from literature that MaxEnt enhances strong peaks and reduces weak peaks^28^ match our results that the strong signal of tyrosine is overestimated while the other four weak signals are severely underestimated. In particular, reconstruction of weak peaks in spectra with large dynamic range are more feasible with CS than with MaxEnt as the latter tends to diminish their peak intensities^27^. Although quantitative accuracy is not significantly different between CS-IRLS and CS-IST, CS-IRLS does show to reconstruct slightly more signals and over all is more accurate and precise. Kazimierczuk *et al*. explained their observed limited performance of IST compared to IRLS in that IST is well suited for spectra with a modest dynamic range while CS-IRLS applies in more demanding cases with high dynamic range as in our case^29^. Clearly, spectral quality does not only depend on the reconstruction technique to compute the NUS spectrum but also on the sampling scheme chosen^11,30,31^. NUS schemes can be divided into on-grid and off-grid sampling. The former is characterized by sampling along the analogous US evolution times, while the latter does not fall on this Cartesian grid^11,24^. Off-grid sampling is applied mostly to spectra of more than two dimensions or requiring particularly narrow peak widths^32^. Examples of sampling schemes with point coordinates not falling on the Cartesian grid are radial sampling, spiral sampling, and concentric ring sampling (CRS)^24,32^. Similar to the on-grid sampling schemes applied in this contribution, burst sampling addresses the aspect of gaps in its sampling. In contrast to sPGS, which minimizes the length of gaps, burst-mode sampling minimizes the number of gaps^24^. Furthermore, beat-matched sampling (BMS) matches the sampling density to the signal envelope analogous to exponentially weighted sampling, but considers in addition the finer details of the predicted time-domain data^11,24,33^. Employing a set of known frequencies the expected time-domain signal is modelled as a sum of exponentially decaying sinusoids and the BMS pattern is adapted to collect data only at the greatest intensities of the modelled signal^33^. However, to our knowledge BMS has so far only been tested with moderate success on synthetic data^33^. As far as our data are concerned, sinusoidal Poisson-gap sampling outperformed unweighted and exponentially weighted sampling particularly in the reconstruction of weak signals.

The variation introduced by the seed number used for initializing the random number generator has been reported to influence the reliability of spectral reconstruction^20^ with sinusoidal Poisson-gap sampling being less affected than unweighted and exponentially weighted sampling^14^. NUS was expected to be more successful on the sparser ^1^H,^1^H-COSY45 than ^1^H,^1^H-TOCSY spectra. Surprisingly, for 75% and 50% NUS ^1^H,^1^H-COSY45 and ^1^H,^1^H-TOCSY are fairly comparable. For the feasibility of NUS, the number of experimental data points acquired relates to the number of signals in the spectrum^34^. The minimum amount of indirect data points required to reliably reconstruct a spectrum is influenced by the signal density and spectral type^20^, and it is advised to choose a more conservative approach when dealing with many signals^20^, crowded spectra^35^ or complex mixtures^36^. When the sensitivity or number of collected indirect data points is too low, NUS spectra are prone to artefacts^37^. A drastic reduction in experimental time by means of NUS is only possible if sensitivity is not limited^38^. Here, the use of 50% NUS did not affect the determination of the urinary levels of metabolites of interest.

To demonstrate the suitability of NUS for a real case study of accelerated metabolite quantification, relevant especially in the context of diabetic nephropathy^39–44^ urinary specimens of apparently healthy volunteers of the GNC cohort were compared to those with chronic kidney disease of the GCKD study. Although the lower limits of quantification (LLOQs) were higher for NUS than US, they were still sufficiently low to determine the urinary levels of metabolites of interest. Overall, there is a good agreement between the data obtained by US and 50% NUS, respectively, as evidenced by the Bland-Altman plots in Figures S14 and S15a. The only exception is the weak tryptophan signal for which larger differences were observed. The Bland-Altman plots comparing the quantitative agreement between 1D ^1^H and 50% NUS spectra (Fig. S13), however, show signal intensity dependent differences predominantly for lactic acid and glutamine, both highly overlapped in 1D ^1^H spectra. As shown in Figure S12a, both US and 50% NUS ^1^H,^1^H COSY45 allow the reliable determination of fold changes for glutamine and lactic acid, therefore, it is safe to assume that the observed variations are due to imperfect signal integration in 1D spectra, which is hampered despite signal deconvolution from extensive signal overlap present in biological matrices such as human urine. Furthermore, the boxplots and *p*-values derived from the NUS and US data are comparable. The similarity of the US and 50% NUS data shows that NUS is suitable for quantifying compounds present at physiological concentrations in complex biological specimens in half the measurement time.

In conclusion, this is the first study to demonstrate that 50% NUS can be applied to the determination of metabolites in real-life biological specimens without a noteworthy drop in the number of reconstructed signals, accuracy, precision, and sensitivity using a combination of sine-weighed Poisson-gap sampling and a compressed sensing approach employing the iterative re-weighted least squares method for the reconstruction of 2D homonuclear ^1^H,^1^H spectra. Together with other advances in instrument design, such as state-of-the-art cryogenic probes, use of 2D NMR spectroscopy in large cohort studies for biomedical applications seems feasible with further reductions in measurement time to about one hour per specimen.

## Methods

### Samples

Six spike-in samples and one blank control were used to assess NUS for the accelerated quantification of urinary metabolites. The initial urine specimen (urine I) from an apparently healthy female volunteer of the German National Cohort (GNC) had a relatively low^45^ creatinine concentration (3.31 ± 0.07 mM) and, thus, an overall low content of solutes. It was utilized to determine the best combination of parameters for the usage of NUS. To address the effect of differences in matrix composition on the finally chosen NUS conditions, a second, analogous spike-in dataset was generated for another urine specimen (urine II) of the GNC cohort^46^ that featured a higher creatinine concentration (20.94 ± 0.27 mM). Further, 28 urine specimens each were randomly chosen from the GNC and the German Chronic Kidney Disease (GCKD) study^47^, respectively.

In course of the GNC study a random sample of the general population is drawn. All study procedures and protocols are accompanied by the external Ethics Advisory Board of the German National Cohort and approvals for all study procedures and protocols were provided by the ethics committees of all corresponding study centres (Helmholtz Centre Munich, Max Delbrueck Centre for Molecular Medicine Berlin Buch, Charité Berlin, German Institute of Human Nutrition Potsdam-Rebruecke, Leibniz Institute of Prevention Research and Epidemiology-BIPS GmbH Bremen, German Diabetes Center Düsseldorf, IUF Leibniz Research Institute for Environmental Medicine Düsseldorf, University Duisburg-Essen, University Freiburg, University Halle-Wittenberg, University Medical Centre Hamburg-Eppendorf, Helmholtz Centre for Infection Research Hannover, University Kiel, University Leipzig, German Cancer Research Center Heidelberg, University Heidelberg, University Münster, University Greifswald, University Regensburg, Cancer Registry Saarland). Written informed consent was obtained from all participants. The study was carried out in accordance with relevant guidelines and regulations.

The German Chronic Kidney Disease (GCKD) study is a prospective cohort study of patients with CKD treated by nephrologists. It was approved by the local ethics committees and registered in the national registry for clinical studies (DRKS 00003971). All study procedures and protocols were approved by the ethics committees of all participating institutions (Friedrich-Alexander-University Erlangen-Nuremberg, Medical Faculty of the Rheinisch-Westfälische Technische Hochschule Aachen, Charité—University Medicine Berlin, Medical Center—University of Freiburg, Medizinische Hochschule Hannover, Medical Faculty of the University of Heidelberg, Friedrich-Schiller-University Jena, Medical Faculty of the Ludwig-Maximilians-University Munich, Medical Faculty of the University of Würzburg). The study was carried out in accordance with relevant guidelines and regulations. Between 2010 and 2012, 5,217 eligible adult patients provided written informed consent and thereafter were enrolled into the study^47^. The characteristics of the subjects investigated are provided in Table S15.

### Sample Preparation

Separate stock solutions for each spike-in dataset of six endogenous urine metabolites^45^, which give overlapping signals in the aromatic and aliphatic regions of 1D ^1^H NMR spectra but are resolved in 2D ^1^H,^1^H TOCSY and ^1^H,^1^H COSY45 spectra (Fig. 1, Supplemental Tables S16 and S17), namely glutamic acid, glutamine, lactic acid, threonine, tryptophan, and tyrosine, all purchased from Sigma-Aldrich, Steinheim, Germany, were prepared in water purified by a PURELAB Plus system [ELGA LabWater, Celle, Germany]. The stocks were each geometrically diluted in six steps so that the added amounts of metabolites represented physiological concentration ranges of 15.6 to 500.0 µM (Table S18)^45^. Further details regarding sample preparation are given in Zacharias *et al*.^48^ and in the supporting material.

### NMR Spectroscopy

1D ^1^H-NOESY, 2D ^1^H,^1^H-TOCSY, and ^1^H,^1^H-COSY45 NMR spectra were acquired at 298 K on an Avance III 600 MHz spectrometer [Bruker BioSpin GmbH, Rheinstetten, Germany] employing a cryogenically cooled triple-resonance (^1^H, ^13^C, ^15^N, ^2^H lock) probe equipped with *z*-gradients and an automatic cooled sample changer. Before measurement, each sample was allowed to equilibrate for 300 s and the probe was automatically locked, tuned, matched, and shimmed. A further description of used NMR parameters can be found in the supporting information. The NUS datasets of the spike-in specimens were either directly measured, being the case for the comparison of R-MDD and CS-IRLS, or generated, as done for all consecutive comparisons, by selecting from a representative US spectrum the respective data points, employing in-house scripts (Supporting Information, pages S44 and 45 and Table S19) specific for each NUS scheme. For measuring the 50% NUS spectra of the cohort samples, a sine-weighted Poisson-gap sampling (sPGS) scheme using the default seed value of the random number generator implemented within TopSpin3.1 was applied. Non-uniformly sampled spectra were reconstructed either with R-MDD^49^, the iterative re-weighted least squares (IRLS) or iterative soft thresholding (IST) algorithm applied as a compressed sensing (CS) approach^29^ or maximum entropy (MaxEnt)^18^, the former two implemented in TopSpin3.1, CS-IST in MestReNova v.12.0.1-20560, and MaxEnt in the Rowland NMR Toolkit (Table S19). Details regarding spectral analysis, evaluation of quantitative results, and statistical data analysis are provided in the supporting information.

### Data availability

The datasets generated during and/or analysed during the current study are available from the corresponding author on reasonable request.

## Electronic supplementary material

Supporting Information
