# Supplementary material for: Systematic Evaluation of Non-Uniform Sampling Parameters in the Targeted Analysis of Urine Metabolites by 1H,1H 2D NMR Spectroscopy
Source: Sci Rep. 2018 Mar 9;8:4249. doi: 10.1038/s41598-018-22541-0 (PMC5844889; doi:10.1038/s41598-018-22541-0)
Supplement: Supplementary file 1 — Supporting Information [file 41598_2018_22541_MOESM1_ESM.docx]

Supporting Information

**Systematic Evaluation of Non-Uniform Sampling Parameters in the Targeted Analysis of Urine Metabolites by ^1^H,^1^H 2D NMR Spectroscopy**

Trixi von Schlippenbach, Peter J. Oefner, Wolfram Gronwald*

Institute of Functional Genomics, University of Regensburg, Am BioPark 9, 93053 Regensburg, Germany

*Corresponding Author

E-mail: wolfram.gronwald@uni-regensburg.de

Accelerated Quantification Spike-Ins Recovery Plots 2

Accelerated Quantification Spike-Ins Tables 13

Fold Changes From All Pairwise Comparisons 25

Application of Accelerated Quantification with NUS to Urinary Specimens of CKD Patients and Healthy Subjects 28

Lower Limits of Quantification (LLOQs) Cohort Study Metabolites 34

Supplemental Materials and Methods 35

Cohort Study Participants 39

Specified Signal Overlap and Quantification 40

Overview Reconstruction Usages 43

Simulation of NUS Spectra 44

References 46

# Accelerated Quantification Spike-Ins Recovery Plots

RECONSTRUCTION ALGORITHMS

**Figure S1.** Signal reconstruction with R-MDD. Influence of the signal reconstruction with R-MDD on the recovery of the relative intensity of a selected cross signal in each spike-in sample and the blank control employing urine I, exemplarily shown for tryptophan (**a**), tyrosine (**b**), lactic acid (**c**), and threonine (**d**). US ^1^H, ^1^H TOCSY spectra (n = 5) indicated by blue bars. NUS ^1^H, ^1^H TOCSY spectra measured with 75%, 50%, and 25% of the linearly sampled data points depicted by green, orange, and grey bars, respectively. Each NUS spectrum was acquired with an exponentially weighted sampling scheme taking three seed values per NUS level. On the x-axis, the spike-in concentration given in micromolar is shown. The intensity ratio of the total cross peak integral scaled to the internal standard TSP of the metabolite signal (mean + SD) obtained with US or NUS to US is plotted on the y-axis.

**Figure S2.** Signal reconstruction with CS-IRLS. Influence of the signal reconstruction with CS-IRLS on the recovery of the relative intensity of a selected cross signal in each spike-in sample and the blank control employing urine I, exemplarily shown for tryptophan (**a**), tyrosine (**b**), lactic acid (**c**), and threonine (**d**). US
^1^H, ^1^H TOCSY spectra (n = 5) indicated by blue bars. NUS ^1^H, ^1^H TOCSY spectra measured with 75%, 50%, and 25% of the linearly sampled data points depicted by green, orange, and grey bars, respectively. Each NUS spectrum was acquired with an exponentially weighted sampling scheme taking three seed values per NUS level. On the x-axis, the spike-in concentration given in micromolar is shown. The intensity ratio of the total cross peak integral scaled to the internal standard TSP of the metabolite signal (mean + SD) obtained with US or NUS to US is plotted on the y-axis.

**Figure S3**. X/Y plots supplementing the corresponding bar charts on signal reconstruction influence. Scatter plots showing the correlation between US (x-axis) and NUS (y-axis) normalised peak volumes when implementing the reconstruction method recursive multidimensional decomposition (R-MDD) or the compressed sensing approach employing the iterative re-weighted least squares method (CS-IRLS) depicted by purple and green data points, respectively. The relative peak intensities, being the intensity ratio of the total cross peak integral of the corresponding metabolite signal scaled to the internal standard TSP, was obtained for US from the mean of five measurements while for NUS with 25% (**a**), 50% (**b**), or 75% (**c**) sampling density. Each data series corresponds to the relative peak intensity of a given metabolite over all spike-in concentrations for the number of observed recoveries (see Table S1) having taken a defined seed value of three.

SAMPLING SCHEMES

**Figure S4.** Use of unweighted sampling. Influence of the unweighted sampling scheme on the recovery of the relative intensity of a selected cross signal of tryptophan (**a**), glutamine (**b**), glutamic acid (**c**), and threonine (**d**) in each spike-in sample and the blank control employing urine I. US ^1^H, ^1^H TOCSY
spectrum (n = 1) indicated by blue bars. NUS ^1^H, ^1^H TOCSY spectra simulated from the US spectrum displayed with 75%, 50%, and 25% of the linearly sampled data points depicted by green, orange, and gray bars, respectively. Each NUS spectrum was constructed with six seed values per NUS level and reconstructed with the compressed sensing approach employing the iterative re-weighted least squares method. On the x-axis, the spike-in concentration given in micromolar is shown. The intensity ratio of the total cross peak integral scaled to the internal standard TSP of the metabolite signal (mean + SD) obtained with US or NUS to US is plotted on the y-axis.

**Figure S5.** Use of exponentially weighted sampling. Influence of the exponentially weighted sampling scheme on the recovery of the relative intensity of a selected cross signal of tryptophan (**a**), glutamine (**b**), glutamic acid (**c**), and threonine (**d**) in each spike-in sample and the blank control employing urine I. US
^1^H, ^1^H TOCSY spectrum (*n* = 1) indicated by blue bars. NUS ^1^H, ^1^H TOCSY spectra simulated from the US spectrum displayed with 75%, 50%, and 25% of the linearly sampled data points depicted by green, orange, and gray bars, respectively. Each NUS spectrum was constructed with six seed values per NUS level and reconstructed with the compressed sensing approach employing the iterative re-weighted least squares method. On the x-axis, the spike-in concentration given in micromolar is shown. The intensity ratio of the total cross peak integral scaled to the internal standard TSP of the metabolite signal (mean + SD) obtained with US or NUS to US is plotted on the y-axis.

**Figure S6.** Use of sinusoidal Poisson-gap sampling. Influence of the sinusoidal Poisson-gap sampling scheme on the recovery of the relative intensity of a selected cross signal of tryptophan (**a**), glutamine (**b**), glutamic acid (**c**), and threonine (**d**) in each spike-in sample and the blank control employing urine I. US
^1^H, ^1^H TOCSY spectrum (*n* = 1) indicated by blue bars. NUS ^1^H, ^1^H TOCSY spectra simulated from the US spectrum displayed with 75%, 50%, and 25% of the linearly sampled data points depicted by green, orange, and gray bars, respectively. Each NUS spectrum was constructed with six seed values per NUS level and reconstructed with the compressed sensing approach employing the iterative re-weighted least squares method. On the x-axis, the spike-in concentration given in micromolar is shown. The intensity ratio of the total cross peak integral scaled to the internal standard TSP of the metabolite signal (mean + SD) obtained with US or NUS to US is plotted on the y-axis.

**Figure S7.** X/Y plots supplementing the corresponding bar charts on sampling scheme influence. Scatter plots showing the correlation between US (x-axis) and NUS (y-axis) normalised peak volumes when implementing the sampling schemes unweighted sampling (UwSa, exponentially weighted sampling (ExSa), or sine-weighted Poisson-gap sampling (sPGS) depicted by purple, green, and red data points, respectively. The relative peak intensities, being the intensity ratio of the total cross peak integral of the corresponding metabolite signal scaled to the internal standard TSP, was obtained for US from the measurement taken for generating simulated NUS spectra while for NUS with 25% (**a**), 50% (**b**), or 75% (**c**) sampling density. Each data series corresponds to the relative peak intensity of a given metabolite over all spike-in concentrations for the number of observed recoveries (see Table S1) having taken a defined seed value of six.

SPECTRA TYPES

**Figure S8.** Comparison of spectra types. Influence of the spectral type on the recovery of the relative intensity of a selected cross signal of tryptophan (**a**), glutamic acid (**b**), and threonine (**c**) in each spike-in sample and the blank control employing urine I. US ^1^H, ^1^H COSY45 spectrum (*n* = 1) indicated by blue bars. NUS spectra simulated from the US spectrum displayed with 75%, 50%, and 25% of the linearly sampled data points depicted by green, orange, and gray bars, respectively. Each NUS spectrum was constructed with a sinusoidal Poisson-gap sampling scheme taking six seed values per NUS level and reconstructed with the compressed sensing approach employing the iterative re-weighted least squares method. On the x-axis, the spike-in concentration given in micromolar is shown. The intensity ratio of the total cross peak integral of the metabolite signal scaled to the internal standard TSP (mean + SD) obtained with US or NUS to US is plotted on the y-axis.

**Figure S9.** X/Y plots supplementing the corresponding bar charts on spectra type influence. Scatter plots showing the correlation between US (x-axis) and NUS (y-axis) normalised peak volumes when implementing ^1^H,^1^H TOCSY or ^1^H,^1^H-COSY45 depicted by purple and green data points, respectively. The relative peak intensities, being the intensity ratio of the total cross peak integral of the corresponding metabolite signal scaled to the internal standard TSP, was obtained for US from the measurement taken for generating simulated NUS spectra while for NUS with 25% (**a**), 50% (**b**), or 75% (**c**) sampling density. Each data series corresponds to the relative peak intensity of a given metabolite over all spike-in concentrations for the number of observed recoveries (see Table S1) having taken a defined seed value of six.

**Figure S10****.** Signal reconstruction with CS-IST. Influence of the signal reconstruction with compressed sensing employing the iterative soft thresholding method on the recovery of the relative intensity of a selected cross signal of tryptophan (**a**), tyrosine (**b**), glutamine (**c**), glutamic acid (**d**), lactic acid (**e**), and threonine (**f**) in each spike-in sample and the blank control employing urine I. US ^1^H,^1^H-COSY45 spectrum (n = 1) indicated by blue bars. NUS spectra simulated from the US spectrum displayed with 50% of the linearly sampled data points depicted by orange bars. Each NUS spectrum was constructed with a sinusoidal Poisson-gap sampling scheme taking six seed values per NUS level. On the x-axis, the spike-in concentration given in micromolar is shown. The intensity ratio of the total cross peak integral of the metabolite signal scaled to the internal standard TSP (mean + SD) obtained with US or NUS to US is plotted on the y-axis.

**Figure S11.** Signal reconstruction with MaxEnt. Influence of the signal reconstruction with maximum entropy on the recovery of the relative intensity of a selected cross signal of tryptophan (**a**), tyrosine (**b**), glutamine (**c**), glutamic acid (**d**), lactic acid (**e**), and threonine (**f**) in each spike-in sample and the blank control employing urine I. US ^1^H,^1^H-TOCSY spectrum (n = 1) indicated by blue bars. NUS spectra simulated from the US spectrum displayed with 50% of the linearly sampled data points depicted by orange bars. Each NUS spectrum was constructed with a sinusoidal Poisson-gap sampling scheme taking three seed values per NUS level. On the x-axis, the spike-in concentration given in micromolar is shown. The intensity ratio of the total cross peak integral of the metabolite signal scaled to the internal standard TSP (mean + SD) obtained with US or NUS to US is plotted on the y-axis.

# Accelerated Quantification Spike-Ins Tables

**Table S1.** Number of observed recoveries observed per parameter from the spike-in data employing urine I. The analysis of reconstruction algorithms and sampling schemes is based on ^1^H,^1^H-TOCSY spectra. To determine the optimal spectra type, the last column contains data from NUS ^1^H,^1^H-COSY45 spectra reconstructed with CS-IRLS and generated with sPGS. Note that for glutamic acid, only five signals were considered as its signal intensities in the blank sample and lowest spike-in concentration were not present or too low for reliable integration even in the US ^1^H-^1^H-TOCSY spectra leading to 40 instead of 42 expected recoveries per NUS level and to 120 expected recoveries over all NUS levels. Abbreviations: R-MDD, recursive multidimensional decomposition; CS-IRLS, compressed sensing approach employing the iterative re-weighted least squares method; USa, unweighted sampling; ExSa, exponentially weighted sampling; sPGS, sine-weighted Poisson-gap sampling.

| NUS level | Reconstruction algorithms | | Sampling schemes | | | Spectra type |
| --- | --- | --- | --- | --- | --- | --- |
|  | R-MDD | CS-IRLS | USa | ExSa | sPGS | ^1^H,^1^H-COSY45 |
| all | 103 | 112 | 98 | 112 | 113 | 118 |
| 75% | 39 | 40 | 33 | 40 | 40 | 40 |
| 50% | 37 | 38 | 34 | 39 | 40 | 40 |
| 25% | 27 | 34 | 31 | 33 | 33 | 38 |

**Table S2.** Comparison of reconstruction algorithms, sampling schemes, and spectra types for the spike-in data of urine I. P-values derived from the comparison of the quantitative performance of NUS utilizing different reconstruction algorithms, sampling schemes or spectra types using the Wilcoxon signed-rank test^a^ or the Friedman test^b^ on ratios (accuracy) and CVs (precision) derived from recoveries over all spike-in metabolites and concentrations either over all NUS levels or per NUS level. Data concerning the reconstruction algorithms and sampling schemes are based on ^1^H,^1^H-TOCSY spectra. Abbreviations:
R-MDD, recursive multidimensional decomposition; compressed sensing approach employing the iterative re-weighted least squares method; USa, unweighted sampling; ExSa, exponentially weighted sampling; sPGS, sine-weighted Poisson-gap sampling; CV, coefficient of variation.

| NUS level | Reconstruction algorithms^a^ | | Sampling schemes^b^ | | Spectra types^a^ | |
| --- | --- | --- | --- | --- | --- | --- |
|  | R-MDD vs. CS-IRLS | | USa vs. ExSa vs. sPGS | | ^1^H,^1^H-TOCSY vs.  ^1^H,^1^H-COSY45 | |
|  | ratio | CV | ratio | CV | ratio | CV |
| all | 4.109e-6 | 0.037 | 2.536e-8 | < 2.2e-16 | 1.075e-4 | 4.585e-8 |
| 75% | 0.032 | 0.100 | 4.027e-7 | 3.851e-9 | 0.150 | 1.162e-6 |
| 50% | 0.164 | 0. 695 | 2.384e-10 | 1.801e-5 | 0.002 | 0.179 |
| 25% | 7.838e-5 | 0.091 | 8.164e-8 | 2.427e-7 | 0.080 | 1.790e-4 |

**Table S3.** Quantitative performance of reconstruction algorithms and sampling schemes from the spike-in data employing urine I. Means of the ratios (accuracy) and CVs (precision) derived from recoveries over all spike-in metabolites and concentrations either over all NUS levels or per NUS level for comparing the quantitative performance of NUS utilizing different reconstruction algorithms or sampling schemes employing ^1^H,^1^H-TOCSY spectra. Abbreviations: R-MDD, recursive multidimensional decomposition; CS-IRLS, compressed sensing approach employing the iterative re-weighted least squares method; USa, unweighted sampling; ExSa, exponentially weighted sampling; sPGS, sine-weighted Poisson-gap sampling; CV, coefficient of variation.

| NUS level | Reconstruction algorithms | | | | | Sampling schemes | | | | | | | | |
| --- | --- | --- | --- | --- | --- | --- | --- | --- | --- | --- | --- | --- | --- | --- |
|  | R-MDD | | CS-IRLS | | | USa | | | ExSa | | | sPGS | | |
|  | ratio | CV | | ratio | CV | | ratio | CV | | ratio | CV | | ratio | CV |
| all | 0.89 | 17.16 | | 0.96 | 12.30 | | 0.97 | 12.06 | | 1.02 | 9.59 | | 1.03 | 5.39 |
| 75% | 0.95 | 8.89 | | 0.98 | 7.23 | | 1.03 | 12.49 | | 1.17 | 4.36 | | 1.06 | 2.56 |
| 50% | 0.94 | 15.13 | | 0.99 | 12.98 | | 1.00 | 11.63 | | 0.92 | 10.87 | | 1.05 | 6.49 |
| 25% | 0.74 | 31.88 | | 0.89 | 17.50 | | 0.88 | 12.06 | | 0.96 | 14.40 | | 0.98 | 7.49 |

**Table S4.** Comparison of sampling schemes from the spike-in data employing urine I. P-values derived from comparing the quantitative performance of NUS utilizing different sampling schemes with each other were obtained having applied the Nemenyi post hoc test on ratios (accuracy) and CVs (precision) derived from recovery plots over all spike-in metabolites and concentrations either over all NUS levels or per NUS level. Data are based on ^1^H,^1^H-TOCSY spectra. Abbreviations: UwSa, unweighted sampling; ExSa, exponentially weighted sampling; sPGS, sine-weighted Poisson-gap sampling.

| NUS level | Sampling schemes | | | | | |
| --- | --- | --- | --- | --- | --- | --- |
|  | ratio | | | CV | | |
|  | USa vs. ExSa | USa vs. sPGS | ExSa vs. sPGS | USa vs. ExSa | USa vs. sPGS | ExSa vs. sPGS |
| all | 3.9e-5 | 1.8e-7 | 0.54 | 0.88 | 4.8e-14 | 2.9e-13 |
| 75% | 4.6e-6 | 0.97 | 1.4e-5 | 0.06526 | 2.3e-9 | 0.00027 |
| 50% | 0.17 | 1.0e-5 | 3.9e-10 | 0.93987 | 0.00034 | 8.2e-5 |
| 25% | 1.8e-5 | 1.8e-5 | 1 | 0.53767 | 0.00053 | 4.6e-6 |

**Table S5.** Quantitative performance of spectra types from the spike-in data employing urine I. Means of the ratios (accuracy) and CVs (precision) over all spike-in metabolites and concentrations either derived from the depicted recoveries either over all NUS levels or per NUS level. Abbreviation: CV, coefficient of variation.

| NUS level | Spectra type | |
| --- | --- | --- |
|  | ^1^H,^1^H-COSY45 | |
|  | ratio | CV |
| all | 0.93 | 8.89 |
| 75% | 1.02 | 5.66 |
| 50% | 0.96 | 7.82 |
| 25% | 0.80 | 13.43 |

**Table S6.** Comparison of NUS levels from the spike-in data employing urine I. P-values derived from comparing the quantitative performance of NUS mutually were obtained having applied the Friedman test^a^ and consecutive Nemenyi post hoc test^b^ on ratios (accuracy) or on CVs (precision) over all spike-in metabolites and concentrations derived from the given recovery plot. Abbreviation: CV, coefficient of variation.

| NUS level | | | | | | | | |
| --- | --- | --- | --- | --- | --- | --- | --- | --- |
| ratio | | | | CV | | | | |
| all NUS levels^a^ | 75% vs. 50% NUS^b^ | 75% vs. 25% NUS^b^ | 50% vs. 25% NUS^b^ | all NUS levels**^a^** | 75% vs. 50% NUS^b^ | 75% vs. 25% NUS^b^ | 50% vs. 25% NUS^b^ | |
| <2.2e-16 | 4.9e-4 | 2.5e-14 | 2.7e-06 | 4.0e-6 | 0.065 | 2.1e-6 | | 0.018 |

**Table S7.** Number of observed recoveries observed per applied supplementary reconstruction algorithm for the spike-in data employing urine I. Data are based on 50% NUS ^1^H,^1^H-COSY45^a^ and ^1^H,^1^H-TOCSY^b^ spectra generated with sine-weighted Poisson-gap sampling. Note that all 42 expected recoveries were considered. Abbreviations: CS-IRLS, compressed sensing approach employing the iterative re-weighted least squares method; CS-IST, compressed sensing approach employing the iterative soft thresholding method; MaxEnt, maximum entropy

| NUS level | Reconstruction algorithms | | |
| --- | --- | --- | --- |
|  | CS-IRLS^a^ | CS-IST^a^ | MaxEnt^b^ |
| 50% | 41 | 40 | 31 |

**Table S8.** Quantitative performance of supplementary reconstruction algorithms for the spike-in data of urine I. Means of the ratios (accuracy) and CVs (precision) derived from recoveries over all spike-in metabolites and concentrations for comparing the quantitative performance of 50% NUS utilizing different supplementary reconstruction algorithms employing ^1^H,^1^H-COSY45^a^ and ^1^H,^1^H-TOCSY^b^ spectra. Abbreviations: CS-IRLS, compressed sensing approach employing the iterative re-weighted least squares method; CS-IST, compressed sensing approach employing the iterative soft thresholding method; MaxEnt, maximum entropy; CV, coefficient of variation.

| NUS level | Reconstruction algorithms | | | | | |
| --- | --- | --- | --- | --- | --- | --- |
|  | CS-IRLS^a^ | | CS-IST^a^ | | MaxEnt^b^ | |
|  | ratio | CV | ratio | CV | ratio | CV |
| 50% | 0.96 | 8.01 | 0.93 | 8.47 | 0.61 | 13.70 |

**Table S9.** Comparison of supplementary reconstruction algorithms for the spike-in data of urine I. *P*-values derived from the comparison of the quantitative performance of NUS utilizing the different supplementary reconstruction algorithms using the Friedman test on ratios (accuracy) and CVs (precision) derived from recoveries over all spike-in metabolites and concentrations. Data are based on 50% NUS ^1^H,^1^H-COSY45^a^ and ^1^H,^1^H-TOCSY^b^ spectra generated with sine-weighted Poisson-gap sampling. Abbreviations: CS-IRLS, compressed sensing approach employing the iterative re-weighted least squares method; CS-IST, compressed sensing approach employing the iterative soft thresholding method; MaxEnt, maximum entropy; CV, coefficient of variation.

| NUS level | Reconstruction algorithms | |
| --- | --- | --- |
|  | CS-IRLS^a^ vs. CS-IST^a^ vs. MaxEnt^b^ | |
|  | **ratio** | **CV** |
| 50% | 2.3e-6 | 0.51 |

**Table S10.** Comparison of supplementary reconstruction algorithms for the spike-in data of urine I. *P*-values derived from comparing the quantitative performance of NUS utilizing different supplementary reconstruction algorithms with each other were obtained having applied the Nemenyi post hoc test on ratios (accuracy) derived from recovery plots over all spike-in metabolites and concentrations. Data are based on 50% NUS ^1^H,^1^H-COSY45^a^ and ^1^H,^1^H-TOCSY^b^ spectra generated with sine-weighted Poisson-gap sampling. Abbreviations: CS-IRLS, compressed sensing approach employing the iterative re-weighted least squares method; CS-IST, compressed sensing approach employing the iterative soft thresholding method; MaxEnt, maximum entropy

| NUS level | Reconstruction algorithms | | |
| --- | --- | --- | --- |
|  | ratio | | |
|  | CS-IRLS^a^ vs. CS-IST^a^ | CS-IRLS^a^ vs. MaxEnt^b^ | CS-IST^a^ vs. MaxEnt^b^ |
| all | 0.52 | 4.7e-6 | 6.1e-4 |

**Table S11.** Concentration-dependent quantitative performance for spike-in metabolites from the spike-in data employing urine I. Ratios (accuracy) and coefficients of variation (precision) derived from recovery plots of 50% NUS ^1^H,^1^H-COSY45 spectra for all added concentrations given in micromolar and the mean or median of all spike-in metabolites, namely tryptophan (Trp), tyrosine (Tyr), glutamine (Gln), glutamic acid (Glu), lactic acid (Lac), and threonine (Thr). Abbreviations: conc., concentration; CV, coefficient of variation.

| Spike-in conc. [µM] | Trp | | | Tyr | | Gln | | | | Glu | | Lac | | | | Thr | |
| --- | --- | --- | --- | --- | --- | --- | --- | --- | --- | --- | --- | --- | --- | --- | --- | --- | --- |
|  | ratio | CV | ratio | | CV | | ratio | CV | ratio | | CV | | ratio | CV | ratio | | CV | |
| 0.0 | 0.76 | 22.29 | 1.02 | | 9.34 | | 0.98 | 7.85 | - | | - | | 0.92 | 13.00 | 1.00 | | 15.68 | |
| 15.6 | 0.85 | 19.42 | 1.08 | | 4.67 | | 0.95 | 1.71 | 0.87 | | 15.81 | | 0.88 | 10.91 | 0.94 | | 14.23 | |
| 31.3 | 0.91 | 13.82 | 1.05 | | 10.48 | | 0.96 | 4.65 | 1.03 | | 16.62 | | 0.87 | 9.35 | 0.84 | | 9.72 | |
| 62.5 | 0.85 | 7.83 | 0.88 | | 14.90 | | 0.98 | 3.40 | 0.97 | | 8.34 | | 1.03 | 3.73 | 0.98 | | 7.14 | |
| 125.0 | 0.92 | 4.21 | 0.93 | | 10.21 | | 1.04 | 3.27 | 0.96 | | 4.44 | | 1.00 | 2.21 | 1.11 | | 11.69 | |
| 250.0 | 0.92 | 9.90 | 1.01 | | 2.05 | | 0.98 | 4.45 | 0.95 | | 5.13 | | 1.00 | 1.35 | 1.04 | | 6.36 | |
| 500.0 | 0.95 | 2.61 | 0.97 | | 7.29 | | 1.01 | 1.40 | 1.02 | | 3.48 | | 0.99 | 1.69 | 0.98 | | 1.82 | |
| median | 0.91 | - | 1.01 | | - | | 0.98 | - | 0.97 | | - | | 0.99 | - | 0.98 | | - | |
| mean | - | 11.44 | - | | 8.42 | | - | 3.82 | - | | 8.97 | | - | 6.03 | - | | 9.52 | |

**Table S12.** Concentration-dependent quantitative performance for spike-in metabolites from the spike-in data employing urine II. Ratios (accuracy) and coefficients of variation (precision) derived from recovery plots of 50% NUS ^1^H,^1^H-COSY45 spectra for all added concentrations given in micromolar and the mean or median of all spike-in metabolites, namely tryptophan (Trp), tyrosine (Tyr), glutamine (Gln), glutamic acid (Glu), lactic acid (Lac), and threonine (Thr). Abbreviations: conc., concentration; CV, coefficient of variation.

| Spike-in conc. [µM] | Trp | | | Tyr | | Gln | | | | Glu | | Lac | | | | Thr | |
| --- | --- | --- | --- | --- | --- | --- | --- | --- | --- | --- | --- | --- | --- | --- | --- | --- | --- |
|  | ratio | CV | ratio | | CV | | ratio | CV | ratio | | CV | | ratio | CV | ratio | | CV | |
| 0.0 | - | - | - | | - | | 0.94 | 3.82 | - | | - | | 0.91 | 10.61 | 0.82 | | 6.62 | |
| 15.6 | 0.76 | 59.96 | 0.89 | | 3.68 | | 0.91 | 6.51 | 0.94 | | 9.65 | | 0.86 | 10.56 | 0.88 | | 8.05 | |
| 31.3 | 1.21 | 33.86 | 0.93 | | 4.95 | | 0.98 | 10.54 | 1.04 | | 6.74 | | 0.90 | 8.12 | 0.88 | | 12.53 | |
| 62.5 | 0.78 | 21.39 | 0.89 | | 3.55 | | 0.92 | 8.25 | 1.03 | | 8.31 | | 1.07 | 8.72 | 0.90 | | 6.58 | |
| 125.0 | 0.87 | 16.54 | 1.02 | | 1.34 | | 0.89 | 6.40 | 1.05 | | 6.58 | | 1.05 | 8.59 | 0.89 | | 6.72 | |
| 250.0 | 0.88 | 10.38 | 1.00 | | 0.90 | | 0.83 | 5.49 | 1.01 | | 1.86 | | 1.01 | 3.55 | 0.90 | | 8.00 | |
| 500.0 | 0.94 | 4.51 | 1.00 | | 2.00 | | 1.05 | 4.96 | 0.96 | | 3.33 | | 1.00 | 2.78 | 0.98 | | 5.34 | |
| median | 0.88 |  | 0.96 | |  | | 0.92 |  | 1.02 | |  | | 1.00 |  | 0.89 | |  | |
| mean |  | 24.44 |  | | 2.73 | |  | 6.57 |  | | 6.08 | |  | 7.56 |  | | 7.71 | |

# Fold Changes From All Pairwise Comparisons

**Figure S12a.** Difference between expected and observed fold changes of each spike-in metabolite from the spike-in data employing urine I obtained with US or with 50% NUS. Here, fold changes of two were considered. For this, the relative signal intensity of a given metabolite in the blank control was subtracted from that in the spike-in samples. With six different spike-in concentration levels, this resulted in five fold changes of two per metabolite and spectrum. These five values were averaged to obtain one value per metabolite and spectrum. As each 50% NUS experiment was executed with six replicates (six seed values), this resulted in six values per metabolite. From this data, for all metabolites the mean ± SD were plotted. Data of the US ^1^H,^1^H-COSY45 spectrum (*n* = 1) are indicated by blue bars, 50% NUS by orange bars.

**Figure S12b.** Difference between expected and observed fold changes of each spike-in metabolite from the spike-in data employing urine I obtained with US or 50% NUS. Here, fold changes from all pairwise comparisons were considered. For this, the relative signal intensity of a given metabolite in the blank control was subtracted from that in the spike-in samples. With six different spike-in concentration levels, this resulted in fifteen fold changes per metabolite and spectrum. These 15 values were averaged to obtain one value per metabolite and spectrum. As each 50% NUS experiment was executed with six replicates (six seed values), this resulted in six values per metabolite. From this data, for all metabolites the mean ± SD was plotted. Data of the US ^1^H,^1^H-COSY45 spectrum (*n* = 1) are indicated by blue bars, 50% NUS by orange bars.

**Figure S12c.** Linear dependency between observed and expected fold changes over all metabolites including all fold changes from two to thirty two from the spike-in data employing urine I. The data was log base 2 transformed and plotted as the mean ± SD. The dashed lines represent the regression lines for the US and 50% NUS data, the respective regression equations and coefficients of determination are given in the figure. Data of the US ^1^H,^1^H-COSY45 spectrum and the 50% NUS spectra are indicated by blue and orange squares.

# Application of Accelerated Quantification with NUS to Urinary Specimens of CKD Patients and Healthy Subjects

**Figure S13.** Agreement between 1D ^1^H-NOESY and 2D ^1^H,^1^H-COSY45 50% NUS data of GCKD and GNC specimens. Bland-Altman plots showing the agreement between absolute metabolite quantification given in millimolar derived from 1D ^1^H-NOESY or NUS ^1^H,^1^H-COSY45 spectra (*n* = 56 per metabolite and spectral type; exceptions see legend Table S13) of selected metabolites, namely creatinine (**a**), hippuric acid (**b**), lactic acid (**c**), D-glucose (**d**), citric acid (**e**), and glutamine (**f**) in urine samples from patients with CKD and healthy subjects (GNC). Pseudouridine was not considered because it was not present as a reference in the compound library of Chenomx NMR Suite 8.2. Note that the glucose level of one GCKD sample yielding a concentration of 26.23 mM (derived from 1D) or 26.58 mM (derived from 50% NUS) was not included in the Bland-Altman plot for reasons of depiction but considered for calculation of the mean ± 1.96*SD. The mean metabolite concentration of both methods is given on the x-axis, the difference between both on the y-axis. The solid line marks the mean difference between both methods, the dashed lines the 95% limits of agreement as mean ± 1.96*SD. Note that in case of strong proteinuria, as present for some of the GCKD specimens (Table S15), both the internal standard TSP as well as some of the metabolites may bind to proteins, potentially leading to inaccuracies in metabolite quantification for these specimens. Note that this effect is independent of the used spectra type and therefore, will not impact comparisons between spectra types.

**Figure S14.** Agreement between 2D ^1^H,^1^H-COSY45 US and 50% NUS data of GCKD and GNC specimens. Bland-Altman plots showing the agreement between absolute metabolite quantification given in millimolar derived from US or NUS ^1^H,^1^H-COSY45 spectra (n = 56 per metabolite and sampling with the following exceptions due to missing values or quantified values below the lower limit of quantification (LLOQ): n_tryptophan_ = 8, n_tyrosine_ = 17, n_threonine_ = 16; for further exceptions see legend Table S13) of selected metabolites, namely creatinine (**a**), hippuric acid (**b**), lactic acid (**c**), D-glucose (**d**), citric acid (**e**), pseudouridine (**f**), tryptophan (**g**), tyrosine (**h**), and threonine (**i**) in urine samples from patients with CKD and healthy subjects (GNC). Note that the glucose level of one GCKD sample yielding a concentration of 26.62 mM (derived from US) or 26.58 mM (derived from 50% NUS) was not included in the Bland-Altman plot for reasons of depiction but considered for calculation of the mean ± 1.96*SD. The mean metabolite concentration of both methods is given on the x-axis, the difference between both on the y-axis. The solid line marks the mean difference between both methods, the dashed lines the 95% limits of agreement as mean ± 1.96*SD. Note that in case of strong proteinuria, as present for some of the GCKD specimens (Table S15), both the internal standard TSP as well as some of the metabolites may bind to proteins, potentially leading to inaccuracies in metabolite quantification for these specimens. Note that this effect is independent of the used spectra type and therefore, will not impact comparisons between spectra types.

**Figure S15.** Absolute concentrations (**a**) of glutamine in urine specimens from CKD subjects and the GNC cohort respectively, in millimolar derived from 50% NUS. Note that in case of strong proteinuria, as present for some of the GCKD specimens (Table S15), both the internal standard TSP as well as some of the metabolites may bind to proteins, potentially leading to inaccuracies in metabolite quantification for these specimens. Note that this effect is independent of the used spectra type and therefore, will not impact comparisons between spectra types. (**b**) or US (**c**) ^1^H,^1^H-COSY45 spectra (*n* = 28 per group and sampling, for exceptions see Table S13). Bland-Altman plots showing the agreement between absolute metabolite quantification given in millimolar derived from both 50% NUS and US spectra. The average metabolite concentration of both methods is given on the x-axis, the difference between the two sampling schemes on the y-axis. The solid line marks the mean difference, the dashed lines the 95% limits of agreement as mean ± 1.96*SD. For the boxplots, glutamine concentrations were normalised against the creatinine concentrations in millimolar. A Mann-Whitney *U*-test was applied to test for significant differences in normalised concentrations between the two groups. Abbreviations: conc, concentration; crea, creatinine. *** *p* ≤ 0.001; for exact *p*-values see Table S13.

**Figure S16.** Quantification of cohort study metabolites with 50% NUS. Absolute quantification of selected metabolites, namely creatinine (**a**), hippuric acid (**b**), lactic acid (**c**), D-glucose (**d**), citric acid (**e**), and pseudouridine (**f**) in urine samples from patients with CKD and healthy subjects (GNC), given on the x-axis, depicted as boxplots. The metabolite concentrations given in micromolar derived from 50% NUS ^1^H,^1^H-COSY45 spectra (n = 28 per group, for exceptions see Table S13; note: n = 7 CKD and n = 5 spectra from healthy specimens contained increased F_1_ ridges, predominantly between 3.20 and 4.10 ppm) were normalised to the corresponding creatinine concentration in millimolar except for creatinine itself in each sample shown on the y-axis. A Mann-Whitney U-test was applied to test for significant differences in normalised concentrations between both groups. Abbreviations: conc, concentration; crea, creatinine. Indications: n.s., not significant; ** p ≤ 0.01, *** p ≤ 0.001, **** p ≤ 0.0001; for exact p-values see Table S13.

**Figure S17.** Quantification of cohort study metabolites with US. Absolute quantification of selected metabolites, namely creatinine (**a**), hippuric acid (**b**), lactic acid (**c**), D-glucose (**d**), citric acid (**e**), and pseudouridine (**f**) in urine samples from patients with CKD and healthy subjects (GNC), given on the x-axis, depicted as boxplots. The metabolite concentrations given in micromolar derived from US ^1^H,^1^H-COSY45 spectra (*n* = 28 per group, for exceptions see Table S13; note: *n* = 7 CKD and *n* = 5 spectra from healthy specimens contained increased F_1_ ridges, predominantly between 3.20 and 4.10 ppm) were normalised to the corresponding creatinine concentration in millimolar except for creatinine itself in each sample shown on the y-axis. A Mann-Whitney *U*-test was applied to test for significant differences in normalised concentrations between both groups. Abbreviations: conc, concentration; crea, creatinine. Indications: n.s., not significant;
** *p* ≤ 0.01, *** *p* ≤ 0.001, **** *p* ≤ 0.0001; for exact *p*-values see Table S13.

**Table S13.** Comparison of US and accelerated quantification with NUS. P-values derived from comparing metabolite levels in the cohort study urine samples from patients with CKD and healthy subjects were obtained having applied the Mann-Whitney U-test on relative intensities per metabolite or over all metabolites in specimens from patients with CKD or healthy subjects, normalised to the corresponding creatinine value in each sample derived from measured US or 50% NUS ^1^H,^1^H-COSY45 spectra
(n = 28 per group and sampling; exceptions due to missing values: ^a^n_GCKD_ = 27).

| Metabolite | ^1^H,^1^H COSY45 US | ^1^H,^1^H COSY45 50% NUS |
| --- | --- | --- |
| Creatinine | 0.7145 | 0.7024 |
| Hippuric acid | 0.4297 | 0.3925 |
| Lactic acid | 0.4773^a^ | 0.4568^a^ |
| D-Glucose | 0.3836 | 0.4203 |
| Citric acid | 1.616e-8 | 8.173e-9 |
| Glutamine | 1.24e-4 | 1.24e-4 |
| Pseudouridine | 2.338e-3 | 1.448e-3 |
| Over all | 0.9236 | 0.9456 |

# Lower Limits of Quantification (LLOQs) Cohort Study Metabolites

**Table S14.** Lower limits of quantification (LLOQs). LLOQs of the quantified metabolite signals in the cohort study urine samples given in micromolar derived from US or 50% NUS ^1^H,^1^H-COSY45 spectra of the calibration samples.

| Metabolite | ^1^H,^1^H COSY45 US [µM] | ^1^H,^1^H COSY45 50% NUS [µM] |  |
| --- | --- | --- | --- |
| Creatinine | 78.13 | 312.50 |  |
| Hippuric acid | 4.88 | 9.77 |  |
| Lactic acid | 9.77 | 9.77 |  |
| D-Glucose | 39.06 | 39.06 |  |
| Citric acid | 4.88 | 9.77 |  |
| Glutamine | 9.77 | 9.77 |  |
| Pseudouridine | 19.53 | 39.06 |  |

# Supplemental Materials and Methods

SAMPLE PREPARATION

For the preparation of the six spike-in samples of both datasets, all six metabolites were added at varying concentrations to both urine aliquots equally, thereby ensuring that each concentration level was used only once per compound (Table S18). For each of these samples, in total 200 µL spike-in compounds were mixed with 200 µL of the corresponding urine. For both datasets, an additional blank sample containing 200 µL purified water and 200 µL of the respective urine was generated. The cohort study samples contained 400 µL urine. To determine LLOQs, calibration samples were prepared. Fourteen calibration levels were generated containing a 400 µL master mix of geometrically diluted stock solutions comprising the selected metabolites over a concentration range from 6.10 × 10^-4^ mM to 5.00 mM with the exception of creatinine and glucose spanning concentrations from 2.44 × 10^-3^ mM to 20.00 mM. Calibration points below 1 mM were measured in triplicates. To this end, 200 µL of 0.1 M phosphate buffer at pH 7.4 containing 3.9 mM boric acid [Merck KGaA, Darmstadt, Germany] to prevent bacterial growth and 50 µL of 0.75% (w/v) TSP in deuterium oxide as internal standard [Sigma-Aldrich, Taufkirchen, Germany] were added to each sample.^1^ All mixtures were transferred to 5-mm i. d. NMR tubes [Bruker BioSpin GmbH, Rheinstetten, Germany or NORELL, Inc., Morganton, NC, USA] and stored at -20°C until measurement.

NMR SPECTROSCOPY

NMR data were gathered using the ICON-NMR suite of TopSpin3.1 [Bruker BioSpin GmbH]. 1D ^1^H-NOESY experiments were conducted as described elsewhere.^1^ For the 2D experiments, solvent suppression was achieved in the ^1^H,^1^H-TOCSY and ^1^H,^1^H-COSY45 spectra by a WATERGATE scheme and by presaturation, respectively. For the US spectra, 2048 × 512 data points were recorded over a total spectral width of 12.07 ppm in ^1^H,^1^H-TOCSY and 13.35 ppm in ^1^H,^1^H-COSY45 with 8 transients per increment after initially applying 32 dummy scans. Each 2D spectrum was acquired with a relaxation delay of 3 s, a mixing time of 0.06 s (^1^H,^1^H-TOCSY) and an acquisition time of 0.14 s for ^1^H,^1^H-TOCSY and 0.12 s for ^1^H,^1^H-COSY45 spectra, resulting in a total experimental time of approximately 3 h and 35 min per 2D experiment.

Three sparse sampling densities, namely 25%, 50%, and 75%, were applied to the spike-in samples employing urine I. The sampling patterns implemented were an unweighted, exponentially weighted, and sine-weighted Poisson-gap sampling, in which the indirect data points were selected randomly to reduce sampling artifacts.^2^ Up to six seed values were set to initialize a random number generator. The exponentially weighted sampling was set so that it matched the exponential *T*_2_ signal decay of the spike-in metabolites.^3^ The equally and exponentially weighted sampling schemes were implemented in TopSpin3.1, the Poisson-gap sampling distribution was generated with the web-based Schedule Generator Version 3.0.^4,5^ The unweighted sampling distributions were generated either with the Stat Trek Random Number Generator^6^ or with TopSpin3.1. The calibration sample set was measured with a ^1^H,^1^H-COSY45 pulse sequence in a uniformly sampled and a 50% non-uniformly sampled manner with the exception of the 50% NUS spectra of tryptophan, tyrosine, and threonine, which were simulated from the corresponding US spectra. For the 50% NUS spectra of the calibration set, a sine-weighted Poisson-gap sampling scheme using the default seed value of the random number generator implemented within TopSpin3.1 was applied.

Phase sensitive ^1^H,^1^H-TOCSY spectra were manually phase corrected, while ^1^H,^1^H-COSY45 spectra were acquired in magnitude mode and therefore required no phase correction. Non-uniformly obtained ^1^H,^1^H-TOCSY spectra were processed with a Hilbert transformation to generate the missing imaginary part from the real part before phase correction. Both types of 2D spectra were semi-automatically baseline corrected excluding the solvent spectral region

Spectral processing was achieved with R-MDD and CS-IRLS in TopSpin 3.1, with CS-IST by means of MestRe Nova^7^ and with MaxEnt by using the Rowland NMR Toolkit (RNMRTK)^8^ shared on the NMRbox platform.^9^ NmrPipe and NmrDraw were used to convert the raw data format as well as to display and analyse the reconstructed data.^10^

SPECTRAL ALNALYSIS

Quantification of signals was done relative to TSP with AMIX 3.9.13 [Bruker BioSpin GmbH]. For relative and absolute quantification of metabolites from the 2D NMR spectra, a single, intense, unambiguous, and well-dispersed cross signal per compound was chosen (Table S17). Absolute concentrations obtained from 2D spectra were calculated manually following the work-flow of MetaboQuant 1.3.^11^ Absolute quantification of 1D ^1^H spectra was carried out with MetaboQuant 1.3 or Chenomx NMR Suite 8.2 [Chenomx Inc., Edmonton, Canada], the latter allows signal deconvolution of overlapping signals. Here, to match the reference compound clusters to the individual resonance peaks of a metabolite, a subtraction line was utilized to adjust the clusters for an optimal fit. Absolute concentrations of creatinine in the spike-in urines were determined from 1D ^1^H spectra (*n* = 5 for urine I, *n* = 3 for urine II) of the blank control sample by MetaboQuant 1.3, taking into account a dilution factor of 2. Levels of selected metabolites in the cohort study samples, namely hippuric acid, pseudouridine, lactic acid, D-glucose, citric acid, and glutamine, were normalised to the corresponding creatinine value in each sample to account for variation due to differences in fluid intake.^12^

EVALUATION OF QUANTITATIVE RESULTS

First, the quantitative performance of NUS compared to US on the spike-in sample set employing urine I was evaluated by plotting the recovery of the relative US signal intensity with NUS, taking into account only signals present in all spectra. Taking one measurement or five technical replicates for the US spectra or three to six different NUS spectra that only differed in their seed values into account, the mean relative intensity of a given metabolite derived from spectra obtained with NUS had to be according to FDA guidelines within 15% of the value of the corresponding US spectrum for accurate quantification and the deviation of the total peak volume in the US and NUS spectra was not to exceed 15% of the CV for precise results.^13^ To address effects of the individual urine matrix on the performance of the selected NUS parameters, the derived fold changes from both spike-in datasets were compared. For determining fold changes in the spike-ins, first the relative signal intensity of a quantified metabolite in the corresponding blank sample was subtracted from that in the associated spike-in sample. For the NUS data, always six different seed values were employed. Next, the compound intensities corresponding to a fold change of two or of two to thirty two were compared pairwise, resulting in a set of up to fifteen fold changes for each dataset. Next, the fold changes were averaged and the difference as well as the linear dependency between expected and observed fold changes, for the latter comparing both spike-in datasets, was depicted in bar charts or as a regression analysis, respectively. The quantitative performance of 50% NUS compared to US of the cohort study samples was conducted via boxplots and Bland-Altman plots, for the latter also comparing 50% NUS to 1D ^1^H spectra.

STATISTICAL DATA ANALYSIS

Statistical analysis was performed with Excel 2013 [Microsoft Corporation, Redmond, WA, USA] and *R* version 3.2.3 in RStudio version 0.99.491 [RStudio, Inc., Boston, MA, USA]. Tests considering the spike-in dataset employing urine I were performed over all concentrations, either over all spike-in metabolites or per metabolite, either over all NUS levels or per NUS level considering accuracy and precision separately as derived from the recovery plots. To test for significant differences in the quantitative performance between implemented variants of given NUS parameters, those signal intensities missing in the NUS spectra were imputed with zero, the respective mean ratios and CVs were calculated from non-imputed data. Normal distribution was tested for with the Shapiro-Wilk test. A Wilcoxon signed-rank test was considered when two and a Friedman test (paired data), Kruskal-Wallis test or one-way ANOVA was implemented when three groups were compared, for the latter furthermore applying a Bartlett test to test for homogeneity of variances. As post hoc tests, either the Nemenyi test or a paired Student’s *t*-test was implemented, the latter after testing for equal variances with an *F*-test. *P*-values derived from statistical comparisons of metabolite total peak volumes between the cohort study samples are based on a Mann-Whitney *U*-test. Statistical significance of a given comparison was considered given a *p*-value of less than or equal to 0.05. In order to calculate the minimum number of samples required from each cohort study to spot trends between them,^1,14^ an a priori statistical power analysis was performed with G*Power version 3.1.9.2.^15^ To this end, ten specimens were randomly selected from each group.

# Cohort Study Participants

**Table S15.** Characteristics of cohort study participants. Baseline and pre-test characteristics of subsets of individuals with chronic kidney disease enrolled in the German Chronic Kidney Disease (GCKD) study or healthy subjects from the German National Cohort (GNC) study, respectively, whose urine samples were measured with the optimized NUS parameters. Abbreviations: ACE-I, angiotensin-converting enzyme inhibitor; ACR, albumin/creatinine ratio; ARB, angiotensin receptor blocker; BMI, body mass index; BP, blood pressure; eGFR_cr_, estimated glomerular filtration rate from serum creatinine; NA, not available.

| Characteristic | Chronic Kidney Disease | Healthy |
| --- | --- | --- |
| sample size, *n* | 28^a^ | 28 |
| men, % | 46.43 | 53.57 |
| Age^b^, years | 58.71 ± 12.09 | 52.61 ± 11.77 |
| BMI^b^, kg/m^2^ | 30.59 ± 6.17 | 25.51 ± 3.39 |
| systolic BP^b^, mmHg | 139.21± 19.36 | 126.93 ± 19.57 |
| diastolic BP^b^, mmHg | 76.93 ± 8.79 | 81.96 ± 12.94 |
| serum creatinine^b^, mM | 0.12 ± 0.03^c^ | 0.09 ± 0.02 (8 NA)^d^ |
| eGFR_cr_^b,e,f^, mL/min per 1.73 m^2^ | 54.40 ± 21.77 | 80.84 ± 18.33 (8 NA) |
| ACR^g^, mg/g | 239.18 (37.61-1068.76) | NA |
| treatment with ACE-I or ARB, % | 89.29 | 10.71 |

^a^ proteinuria classification: < 30 mg/L (*n* = 10), 30-300 mg/L (*n* = 6), > 300 mg/L (*n* = 12)

^b^ mean ± SD

^c^ *n* = 1 quantified relative to 2.05 mM spiked-in formic acid with Chenomx NMR Suite 8.2 from a 1D
^1^H-NOESY spectrum of the corresponding plasma sample

^d^ determined with MetaboQuant 1.3 from 1D ^1^H-NOESY spectra of the corresponding serum samples

^e^ estimated using the CKD-EPI equation^16^ without specifying for the black race

^f^ *n*_GCKD_ = 1 and *n*_GNC_ = 20 calculated from given or determined serum creatinine concentrations

^g^ median (interquartile range)

# Specified Signal Overlap and Quantification

**Table S16.** Presence of overlap in 1D ^1^H spectra. ^1^H chemical shifts^17^ (for pseudouridine H1 according to AMIX-viewer 3.9.13) in ppm of mutually overlapped spike-in and cohort study metabolite signals.

| Metabolite | Signal assignment | ^1^H chemical shift [ppm] | Overlap with |
| --- | --- | --- | --- |
| Tryptophan | H10 | 7.19 | tyrosine |
| Tyrosine | H5 H9 | 7.17 | tryptophan |
| Glutamine | H2 | 3.77 | glutamic acid, D-glucose, pseudouridine |
| Glutamine | H4A|B | 2.45 | glutamic acid, citric acid |
| Glutamine | H3A|B | 2.13 | glutamic acid |
| Glutamic acid | H2 | 3.75 | glutamine |
| Glutamic acid | H4B | 2.34 | glutamine |
| Glutamic acid | H3A | 2.12 | glutamine |
| Glutamic acid | H3B | 2.04 | glutamine |
| Lactic acid | H3A|B|C | 1.32 | threonine |
| Lactic acid | H2 | 4.10 | pseudouridine |
| Threonine | H4A|B|C | 1.32 | lactic acid |
| Creatinine | H2A|B | 4.05 | pseudouridine |
| Hippuric acid | H8 | 7.62 | pseudouridine |
| Hippuric acid | H2A|B | 3.96 | pseudouridine, D-glucose |
| D-Glucose | H1β | 4.63 | pseudouridine |
| D-Glucose | H6Bβ | 3.89 | hippuric acid, pseudouridine |
| D-Glucose | H4 H6α | 3.82 | pseudouridine |
| D-Glucose | H6Bα H6Aβ H3α | 3.73 | glutamine, pseudouridine |
| Citric acid | H2A H4A | 2.51 | glutamine |
| Pseudouridine | H12 | 7.66 | hippuric acid |
| Pseudouridine | H1 | 4.69 | D-glucose |
| Pseudouridine | H3 | 4.14 | lactic acid |
| Pseudouridine | H4 | 4.01 | creatinine, hippuric acid |
| Pseudouridine | H5A | 3.84 | D-glucose |
| Pseudouridine | H5B | 3.72 | D-glucose, glutamine |

**Table S17.** 2D signal assignments. ^1^H,^1^H-TOCSY and ^1^H,^1^H-COSY45 chemical shifts^17^ in ppm of metabolite signals used for quantification. Abbreviations: F1, indirect spectral dimension; F2, direct spectral dimension.

| Metabolite | ^1^H,^1^H-TOCSY | | ^1^H,^1^H-COSY45 | |
| --- | --- | --- | --- | --- |
|  | Signal assignment | ^1^H chemical shift in F1/F2 [ppm] | Signal assignment | ^1^H chemical shift in F1/F2 [ppm] |
| Tryptophan | H10/H9 | 7.19/7.72 | H3B/H2 | 3.29/4.03 |
| Tyrosine | H6 H8/H5 H9 | 6.67/7.04 | H6 H8/H5 H9 | 6.67/7.04 |
| Glutamine | H3A|B/H4A|B | 2.13/2.45 | H3A|B/H4A|B | 2.13/2.45 |
| Glutamic acid | H4A|B/H2 | 2.34/3.76 | H3A|B/H4A|B | 2.09/2.34 |
| Lactic acid | H3A|B|C/H2 | 1.32/4.11 | H3A|B|C/H2 | 1.32/4.11 |
| Threonine | H4A|B|C/H2 | 1.32/3.57 | H4A|B|C/H3 | 1.32/4.24 |
| Creatinine | - | - | H6A|B|C/H2A|B | 3.03/4.05 |
| Hippuric acid | - | - | H7 H9/H6 H10 | 7.54/7.82 |
| D-Glucose | - | - | H1β/H2β | 4.63/3.23 |
| Citric acid | - | - | H2A H4A/H2B H4B | 2.53/2.63 |
| Pseudouridine | - | - | H3/H2 | 4.14/4.29 |

**Table S18.** Spike-in sample concentrations equivalent in both spike-in datasets. Metabolite concentrations per spike-in sample given in millimolar.

| Metabolite | sample | | | | | | |
| --- | --- | --- | --- | --- | --- | --- | --- |
|  | blank | 1 | 2 | 3 | 4 | 5 | 6 |
| Tryptophan | - | 500 | 15.6 | 31.3 | 62.5 | 125 | 250 |
| Tyrosine | - | 250 | 500 | 125 | 62.5 | 31.3 | 15.6 |
| Glutamine | - | 62.5 | 125 | 250 | 500 | 15.6 | 31.3 |
| Glutamic acid | - | 125 | 250 | 500 | 15.6 | 31.3 | 62.5 |
| Lactic acid | - | 31.3 | 62.5 | 125 | 250 | 500 | 15.6 |
| Threonine | - | 15.6 | 31.3 | 62.5 | 125 | 250 | 500 |

# Overview Reconstruction Usages

**Table S19.** Reconstruction algorithm usages. Overview of the reconstruction algorithm usages on measured or simulated NUS ^1^H,^1^H-TOCSY and ^1^H,^1^H-COSY45 spectra according to the sampling scheme applied. Abbreviations: USa, unweighted sampling; ExSa, exponentially weighted sampling; sPGS, sine-weighted Poisson-gap sampling; R-MDD, recursive multidimensional decomposition; CS-IRLS, compressed sensing approach employing the iterative re-weighted least squares method; MaxEnt, maximum entropy; CS-IST, compressed sensing approach employing the iterative soft thresholding method.

| Sampling scheme | ^1^H,^1^H-TOCSY NUS | | ^1^H,^1^H-COSY45 NUS | |
| --- | --- | --- | --- | --- |
|  | measured | simulated | measured | simulated |
| USa | - | CS-IRLS | - | - |
| ExSa | R-MDD, CS-IRLS | CS-IRLS | - | - |
| sPGS | - | CS-IRLS, MaxEnt | CS-IRLS | CS-IRLS, CS-IST |

# Simulation of NUS Spectra

For generating simulated NUS spectra, the TopSpin3.1 AU programs “splitser” and “fidtoser” were modified accordingly. The “splitser” command was adapted to extract from an acquired US spectrum individual FIDs according to the sampling density, sampling scheme, and seed value intended. The adapted “fidtoser” command writes the extracted FIDs to a new ser file matching the NUSLIST point schedule to give the simulated NUS spectrum. The NUSLIST comprises one column per indirect dimension containing the data point to be considered, being complex for ^1^H,^1^H-TOCSY spectra, starting with the indices 0 adjusted to the sampled NUS points of the simulated NUS spectrum.

The codes for the adapted “splitser” and “fidtoser” command are exemplarily shown below, comprising the 128 indirect data points corresponding to a 25% NUS dataset with sine-weighted Poisson-gap sampling given the default seed value of TopSpin3.1.

modified “splitser” schedule:

int td;

GETCURDATA

FETCHPAR1S("TD",&td)

int a[128]={ 1,2,3,4,5,6,7,8,9,10,11,12,13,14,15,16,19,20,23,24,25,

26,27,28,29,30,35,36,41,42,47,48,49,50,55,56,61,62,65,66,69,70,71,

72,75,76,79,80,87,88,91,92,93,94,99,100,101,102,107,108,111,112,

117,118,125,126,131,132,141,142,151,152,165,166,171,172,177,178,

179,180,191,192,195,196,205,206,225,226,237,238,243,244,255,256,

263,264,279,280,301,302,317,318,329,330,343,344,357,358,365,366,

379,380,397,398,405,406,417,418,433,434,459,460,473,474,487,488,

509,510};

i1=0;

TIMES(128)

RSER(a[i1],a[i1],1);

i1 ++;

END

QUITMSG("--- splitser finished ---")

modified “fidtoser” schedule

int a[128]={ 1,2,3,4,5,6,7,8,9,10,11,12,13,14,15,16,19,20,23,24,25,

26,27,28,29,30,35,36,41,42,47,48,49,50,55,56,61,62,65,66,69,70,71,

72,75,76,79,80,87,88,91,92,93,94,99,100,101,102,107,108,111,112,

117,118,125,126,131,132,141,142,151,152,165,166,171,172,177,178,

179,180,191,192,195,196,205,206,225,226,237,238,243,244,255,256,

263,264,279,280,301,302,317,318,329,330,343,344,357,358,365,366,

379,380,397,398,405,406,417,418,433,434,459,460,473,474,487,488,

509,510};

char nm1[PATH_MAX];

int ne = 128;

int proc1 = 1;

strcpy(nm1, name);

GETSTRING("Enter name of 1D series:", nm1)

USECURPARS

int i=0;

TIMES(ne)

WSER(loopcount1+1, nm1, a[i], proc1, disk, user)

i += 1;

END

QUIT

# References

1. Zacharias, H. U. *et al.* Current Experimental, Bioinformatic and Statistical Methods used in NMR Based Metabolomics. *Curr. Metabolomics* **1**, 253-268 (2013).

2. Hoch, J. C., Maciejewski, M. W. & Filipovic, B. Randomization improves sparse sampling in multidimensional NMR. *J. Magn. Reson.* **193**, 317-320 (2008).

3. Rai, R. K., Tripathi, P. & Sinha, N. Quantification of Metabolites from Two-Dimensional Nuclear Magnetic Resonance Spectroscopy: Application to Human Urine Samples. *Anal. Chem.* **81**, 10232-10238 (2009).

4. Gerhard Wagner Lab. Schedule Generator Version 3.0. *Harvard Medical School* http://gwagner.med.harvard.edu/intranet/hmsIST/gensched_new.html (2013-14).

5. Hyberts, S. G., Milbradt, A. G., Wagner, A. B., Arthanari, H. & Wagner, G. Application of iterative soft thresholding for fast reconstruction of NMR data non-uniformly sampled with multidimensional Poisson Gap scheduling. *J. Biomol. NMR* **52**, 315-327 (2012).

6. Random Number Generator. *Stat Trek* http://stattrek.com/statistics/random-number-generator.aspx (2017).

7. Willcott, M. R. MestRe Nova. *JACS* **131**, 13180-13180 (2009).

8. Hoch, J. C. & Stern, A. S. *NMR Data Processing* (Wiley, 1996).

9. Maciejewski, M. W. *et al.* NMRbox: A Resource for Biomolecular NMR Computation. *Biophys. J.* **112**, 1529-1534 (2017).

10. Delaglio, F. *et al.* NMRPipe: A multidimensional spectral processing system based on UNIX pipes. *J. Biomol. NMR* **6**, 277-293 (1995).

11. Klein, M. S., Oefner, P. J. & Gronwald, W. MetaboQuant: a tool combining individual peak calibration and outlier detection for accurate metabolite quantification in 1D ^1^H and ^1^H-^13^C HSQC NMR spectra. *BioTechniques* **54**, 251-256 (2013).

12. Barrios, C., Spector, T. D. & Menni, C. Blood, urine and faecal metabolite profiles in the study of adult renal disease. *Arch. Biochem. Biophys.* **589**, 81-92 (2016).

13. U. S. Department of Health and Human Services. Guidance for Industry: Bioanalytical Method Validation. *Center for Drug Evaluation and Research (CDER), Center for Veterinary Medicine (CVM)* https://www.fda.gov/downloads/Drugs/Guidance/ucm070107.pdf (2001).

14. Beckonert, O. *et al.* Metabolic profiling, metabolomic and metabonomic procedures for NMR spectroscopy of urine, plasma, serum and tissue extracts. *Nat. Protoc.* **2**, 2692-2703 (2007).

15. Faul, F., Erdfelder, E., Buchner, A. & Lang, A.-G. Statistical power analyses using G*Power 3.1: Tests for correlation and regression analyses. *Behav. Res. Methods* **41**, 1149-1160 (2009).

16. Levey, A. S. *et al.* A New Equation to Estimate Glomerular Filtration Rate. *Ann. Intern. Med.* **150**, 604-613 (2009).

17. Wishart Research Group. HMDB Version 3.0. *The Human Metabolome Database* http://www.hmdb.ca (2012).
